# Metabolism of 3-Chlorobiphenyl (PCB 2) in a Human-Relevant Cell Line: Evidence of Dechlorinated Metabolites

**DOI:** 10.1021/acs.est.2c03687

**Published:** 2022-08-22

**Authors:** Chun-Yun Zhang, Xueshu Li, Susanne Flor, Patricia Ruiz, Anneli Kruve, Gabriele Ludewig, Hans-Joachim Lehmler

**Affiliations:** † Hubei Key Laboratory of Regional Development and Environmental Response, Faculty of Resources and Environmental Science, Hubei University, Wuhan 430062, China; ‡ Department of Occupational and Environmental Health, The University of Iowa, Iowa City, Iowa 52242, United States; § Office of Innovation and Analytics, Simulation Science Section, Agency for Toxic Substances and Disease Registry, Atlanta, Georgia 30333, United States; ∥ Department of Materials and Environmental Chemistry, Stockholm University, Svante Arrhenius Väg 16, 10691 Stockholm, Sweden

**Keywords:** ADMET predictor, PCB 2 metabolites, HepG2 cells, MetaDrug, Nt-HRMS, dechlorination, untargeted metabolomics

## Abstract

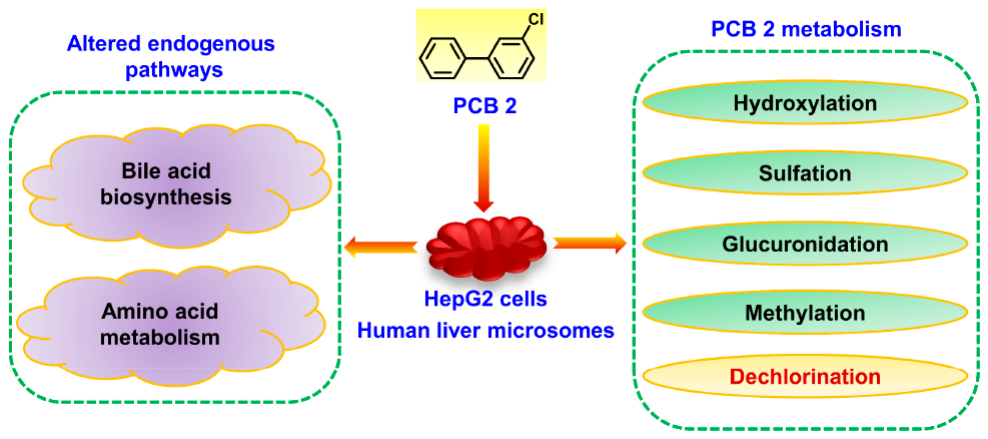

Lower chlorinated polychlorinated biphenyls (LC-PCBs) and their metabolites make up a class of environmental pollutants implicated in a range of adverse outcomes in humans; however, the metabolism of LC-PCBs in human models has received little attention. Here we characterize the metabolism of PCB 2 (3-chlorobiphenyl), an environmentally relevant LC-PCB congener, in HepG2 cells with *in silico* prediction and nontarget high-resolution mass spectrometry. Twenty PCB 2 metabolites belonging to 13 metabolite classes, including five dechlorinated metabolite classes, were identified in the cell culture media from HepG2 cells exposed for 24 h to 10 μM or 3.6 nM PCB 2. The PCB 2 metabolite profiles differed from the monochlorinated metabolite profiles identified in samples from an earlier study with PCB 11 (3,3′-dichlorobiphenyl) under identical experimental conditions. A dechlorinated dihydroxylated metabolite was also detected in human liver microsomal incubations with monohydroxylated PCB 2 metabolites but not PCB 2. These findings demonstrate that the metabolism of LC-PCBs in human-relevant models involves the formation of dechlorination products. In addition, untargeted metabolomic analyses revealed an altered bile acid biosynthesis in HepG2 cells. Our results indicate the need to study the disposition and toxicity of complex PCB 2 metabolites, including novel dechlorinated metabolites, in human-relevant models.

## Abstract 2

Dechlorinated polychlorinated biphenyl (PCB) metabolites formed by HepG2 cells make up a new class of PCB metabolites relevant to human health.

## Introduction

Polychlorinated biphenyls (PCBs) are detectable in most environmental matrices, although their production was banned more than half a century ago.^1−7^ Human exposure to PCBs occurs dermally, through the diet, and by inhalation^8−10^ and can cause adverse health effects, for example, cancer and neurotoxicity.^11−14^ Current human exposure to PCBs by inhalation is comparable to dietary exposure in the United States.^9^ PCB 2 (3-chlorobiphenyl) is a component of lower chlorinated commercial PCB mixtures, such as Aroclor 1221 and Aroclor 1232.^15^ It is also a product of biodegradation of higher chlorinated PCBs by microorganisms.^16,17^ PCB 2 is present in outdoor air^18,19^ and human blood,^20^ thus representing a hazardous material that has received little attention to date.

Cytochrome P450 (P450) enzymes oxidize lower chlorinated PCBs (LC-PCBs) to hydroxylated metabolites (OH-PCBs). These OH-PCBs can be sulfated, glucuronidated, or further oxidized, resulting in complex metabolite mixtures.^21^ For example, PCB 2 and PCB 3 (4-chlorobiphenyl) are oxidized by rat P450 enzymes to OH-PCBs.^22,23^ OH-PCB 3 metabolites and their corresponding sulfate conjugates are also observed in rats exposed to PCB 3.^24−26^ Another LC-PCB congener, PCB 11 (3,3′-dichlorobiphenyl), is oxidized at the *para* position and further conjugated to sulfate and glucuronide metabolites in rats.^27,28^ HepG2 cells metabolize PCB 3 and PCB 11 to complex mixtures of metabolites, including catechol-derived methoxylated metabolites.^29,30^ In addition, dechlorinated OH-PCB metabolites have been reported in animal studies;^31−34^ however, it is unknown if dechlorination reactions can occur in humans.

Several studies have investigated the toxicity of PCB 2 hydroquinone and quinone metabolites. Importantly, these PCB 2 metabolites can covalently bind with DNA.^35^ A PCB 2 quinone, 3′-chloro-biphenyl-2,5-dione, inhibits human topoisomerase.^36^ PCB 2 hydroquinone and the corresponding quinone cause oxidative DNA damage.^37−39^ The PCB 2 hydroquinone inhibits cell cycle progression and induces polyploidization in V79 cells in culture.^40^ Analogously, reactive PCB 3 metabolites are linked to genotoxic effects.^39−45^ PCB 3 and its metabolites are initiators of liver carcinogenesis *in vivo*.^46,47^ In addition, metabolites of lower chlorinated PCBs are potential endocrine-disrupting chemicals.^48−50^

The available evidence indicates that PCB 2 and its metabolites are environmental hazards; however, the metabolism of PCB 2 has not been studied in humans. Here, we characterized the metabolism of PCB 2 with HepG2 cells in culture and human liver microsomes (HLMs) using nontarget high-resolution mass spectrometry (Nt-HRMS). We observed that PCB 2 undergoes biotransformation to hydroxylated, sulfated, glucuronidated, or methoxylated metabolites in human-relevant models, with PCB 2 catechol-derived metabolites playing a central role in PCB 2 bioactivation. Importantly, our results provide compelling evidence that HepG2 cells form dechlorinated PCB 2 metabolites, a PCB metabolic pathway that has not been reported previously in humans. Also, PCB 2 exposure alters bile acid biosynthesis and amino acid metabolism in cells in culture, suggesting novel mechanisms by which lower chlorinated PCBs and their (dechlorinated) metabolites cause adverse outcomes in humans and other mammals.

## Experimental Section

### Chemicals and Other Materials

PCB 2 (purity of >99%) was purchased from AccuStandard, Inc. (New Haven, CT). This commercial product contained 775 ng of biphenyl (BP)/mg of PCB 2 (for the quantification of BP in this PCB 2 batch, see the Supporting Information). 4-Chloro-3-hydroxy-biphenyl (3-OH-PCB 3), 3-chloro-4-hydroxy-biphenyl (4-OH-PCB 2), 4′-chloro-3′-fluoro-4-sulfooxy-biphenyl (3-F,4′-PCB 3 sulfate), and 4′-chloro-3′-fluoro-4-hydroxy-biphenyl (3-F,4′-OH-PCB 3) used as test compounds or internal standards were synthesized and authenticated previously.^26,51^ The cell culture supplies were obtained from Thermo Fisher Scientific (Radnor, PA). The HLMs (pool of 50, mixed gender) were purchased from Xenotech (Lenexa, KS).

### Exposure of HepG2 Cells to PCB 2

The metabolism of PCB 2 was studied using HepG2 cells. This cell line is inexpensive to maintain and is widely used in metabolic studies of environmental pollutants.^52^ HepG2 cells are an ideal model for initial studies of the metabolism of LC-PCBs. The metabolism of LC-PCBs in these cells is slower because of the lower level of expression of drug-processing enzymes in HepG2 cells than in primary hepatocytes.^29^

For information about the source and maintenance of the HepG2 cells, see the Supporting Information. For the metabolic studies, HepG2 cells were seeded in three biological replicates into six-well plates (6 × 10^6^ cells/well) with 3 mL of complete minimum essential medium.^29,30^ Cells were allowed to attach for 48 h, followed by exposure to concentrations of PCB 2 (3.6 nM or 10 μM with 0.1% DMSO) for 24 h in exposure medium supplemented with 4.5 mM d-glucose (3 mL/well). A high concentration of 10 μM and an exposure time of 24 h were selected to ensure the robust detection of PCB 2 metabolites.^29,30^ The concentration of 3.6 nM PCB 2 was used because it is equivalent to the concentration of the PCB 2 impurity in our earlier PCB 11 metabolic study (the PCB 11 used in the earlier study contained 307 ng of PCB 2/mg of PCB 11).^29^ On the basis of our cytotoxicity studies with other PCB congeners,^29,30,53^ HepG2 cells will maintain their metabolic function and viability at these concentrations. Control samples were exposed to the exposure medium containing 0.1% DMSO. The medium samples were harvested 24 h after exposure and stored at −80 °C.

### Incubation of HLMs with PCB 2 and Its Hydroxylated Metabolites

As described previously,^54−56^ 16 mL of an incubation solution containing phosphate buffer (1 M, pH 7.4) with magnesium chloride (3 mM), microsomes (0.1 mg of microsomal protein/mL), and NADPH (0.5 mM) was preincubated at 37 °C for 5 min. PCB 2 (final concentration of 3.6 nM or 10 μM), 4-OH-PCB 2 (final concentration of 10 μM), or 3-OH-PCB 3 (final concentration of 10 μM, a possible 1,2-shift metabolite of PCB 2) in DMSO [0.1% (v/v)] was added to start the reactions. Aliquots (3 mL) were collected at 0, 0.5, 2.5, 5, and 15 min, added to ice-cold formic acid [400 μL, 10% (v/v)], and heated at 110 °C for 10 min before extraction. No PCB metabolites were detected in control incubations with inactive microsomes or in incubations without microsomes, NADPH, or test compounds.

### Extraction of PCB 2 Metabolites

PCB 2 metabolites were extracted from cell culture media or HLM incubations with the QuEChERS method. For cell culture media, the extraction of PCB 2 metabolites followed a published procedure.^29^ An analogous procedure with minor modifications was used for HLM samples. For details regarding the extraction of PCB 2 metabolites from both matrices, see the Supporting Information. Cell pellets were not analyzed because the metabolite levels in the cell pellet are lower than those in the cell culture medium.^29,30,57^

### Liquid Chromatography-High-Resolution Mass Spectrometry (LC-HRMS)

The presence of PCB 2 metabolites in cell culture media was initially assessed with an ultraperformance liquid chromatograph (Waters Acquity UPLC, Waters, Milford, MS) coupled with a quadrupole time-of-flight mass spectrometer (LC-QTof MS, Waters Q-Tof Premier).^29^ For a summary of metabolites identified in this analysis, see Table S1.

Samples were subsequently analyzed with an ultra-high-performance liquid chromatograph (Ultimate 3000 UHPLC+ Focused, Thermo Fisher Scientific, Waltham, MA) equipped with an Acquity UPLC BEH C18 column (Waters; 2.1 mm inner diameter, 100 mm length, 1.7 μM particle size) and coupled with a Q Exactive Hybrid Quadrupole-Orbitrap mass spectrometer (LC-Orbitrap MS, Thermo Fisher) at the Center of Mass Spectrometry and Proteomics at the University of Minnesota (Minneapolis, MN) or a Q-Executive Orbitrap Mass Spectrometer (Thermo Fisher Scientific) with a Vanquish Flex ultra-high-performance liquid chromatograph (Thermo Fisher Scientific) at the High-Resolution Mass Spectrometry Facility of The University of Iowa. The instrument operating conditions were the same as those reported previously.^29,30^ For details regarding LC-HRMS analysis and quality assurance/quality control, see the Supporting Information. Putative PCB 2 metabolites were identified as explained in the Supporting Information, and their experimental MS data matched with the theoretical accurate mass, isotopic pattern, and featured MS/MS product ions.^29^ For representative MS and MS/MS spectra, see Figures S1–S13.

### Calculation of Molecular Response-Independent (MRI) PCB 2 Metabolite Profiles

The ionization efficiency of different isomers of the PCB 2 metabolites was predicted from the PaDEL descriptors with random forest regression (Figure S14)^58^ and used to calculate the MRI profiles of the PCB 2 metabolites. The molecule ion and, where applicable, the fragment ion of each PCB 2 metabolite in the full scan data were integrated from the extracted ion chromatograms with a mass window of 10 ppm. The peak areas were summed for each metabolite class and normalized with one of the internal standards (IS, i.e., 3-F,4′-PCB 3 sulfate) to obtain the normalized intensity from the equation normalized intensity = raw intensity of metabolite/raw intensity of IS × scale factor of 1000. The predicted ionization efficiencies of each PCB 2 metabolite class were averaged for different isomers and used to correct the PCB 2 metabolite intensities (i.e., MRI intensity = normalized intensity/ionization efficiency). The MRI PCB 2 metabolite profiles were calculated as MRI percentage = metabolite MRI intensity/total MRI intensity.

### Metabolomics Analysis

Metabolomics analysis was performed in R (version 3.6.3) as described with minor modifications.^29^ Briefly, feature data (i.e., *m*/*z*, retention time, and intensity of each metabolite) were extracted with *apLCMS*(59) and *xMSanalyzer*.^60^ Univariate analyses comparing exposed and control groups were performed with the *limma* test^61^ with false discovery rate (FDR) correction.^62^ A linear regression model was employed to identify features that showed significant concentration-dependent changes (*p* < 0.05). Pathway analysis was carried out with *mummichog* (version 2.0.6),^29,63^ and features were annotated with the Human Metabolome DataBase (HMDB)^64^ and the Kyoto Encyclopedia of Genes and Genomes (KEGG)^65^ using *xMSannotator*.^66^

## Results and Discussion

### Metabolism of PCB 2 in HepG2 Cells

Nt-HRMS analyses of the cell culture medium collected after exposure for 24 h, in conjunction with the candidate screening list developed with ADMET Predictor and MetaDrug (see the Supporting Information and Tables S2 and S3), revealed the formation of complex mixtures of oxygenated PCB 2 metabolites. The identified metabolites included mono- and dihydroxylated PCB 2 metabolites and the corresponding sulfates and glucuronides (classes 1 and 2). We also observed methoxylated metabolites (class 3) consistent with our studies with PCB 3 and PCB 11.^29,30^ Importantly, putative dechlorinated metabolites (class 4) were detected, suggesting for the first time that some LC-PCBs may undergo dechlorination reactions in humans. Similar to previous studies,^29,30^ we did not detect PCB 2 quinone metabolites or their derivatives, such as glutathiylated biphenyl quinones (dechlorinated metabolite) or glutathiylated PCB 2 hydroquinones.^67^

#### Identification of Monohydroxylated PCB 2 Metabolites and Their Conjugates

We identified one monohydroxylated PCB 2 (class 1.1), one PCB 2 sulfate (class 1.2), and two PCB 2 glucuronide metabolites (class 1.3) (Table 1). As with PCB 3 and PCB 11,^29,30^ the OH-PCB 2 metabolite is likely *para*-hydroxylated; however, the hydroxylated phenyl ring of PCB 2 is unknown. Studies with rat liver P450 enzymes or microsomes suggest that chlorobiphenyls are preferentially metabolized in the lower chlorinated ring.^22,23^ A second OH-PCB 2 isomer was formed transiently, as indicated by the presence of two glucuronide conjugates in a 7:1 ratio. We did not detect PCB 2 dihydrodiols, metabolites that are formed by rat P450 enzymes,^68,69^ or PCB 2 glutathione adducts, consistent with our studies with PCB 3 and PCB 11.^29,30^ The class 1 metabolites of PCB 2 formed by HepG2 cells are in agreement with our computational predictions and earlier metabolic studies with LC-PCBs. For example, the class 1 metabolites of PCB 3 and PCB 11 were detected *in vitro*,^23,29,30^ in animal disposition,^25,26,28,70^ and in human biomonitoring studies.^71^

**Table 1:** Several PCB 2 Metabolite Classes Were Detected by LC-Orbitrap MS Analysis in Medium from HepG2 Cells Exposed to 10 μM or 3.6 nM PCB 2^a^

				MRI or normalized intensity^c^		accurate mass difference^e^ (ppm)			
class	metabolite	retention time^b^ (min)	formula	high	low^d^	high	low^d^	MS^2^ (Da)	confidence level^f^
1.1	OH-PCB 2	5.43	C_12_H_8_ClO^–^	50 ± 27	2 ± 1	2.9	3.5	174.99, 167.05	2
1.2	PCB 2 sulfate	4.22	C_12_H_8_ClSO_4_^–^	80 ± 9	16.2 ± 0.8	1.9	2.0	203.03	2
1.3	PCB 2 glucuronide	3.68	C_18_H_16_ClO_7_^–^	3.2 ± 0.8	0.05 ± 0.01	1.6	1.1	203.03	2
		3.78		0.44 ± 0.07	ND	1.9	ND	203.03	2
2.1	OH-PCB 2 sulfate	3.56	C_12_H_8_ClSO_5_^–^	1.8 ± 0.3	0.31 ± 0.04	2.0	2.1		3
		4.24		0.66 ± 0.02	0.30 ± 0.04	2.0	2.0		3
3.1	MeO-OH-PCB 2	5.72	C_13_H_10_ClO_2_^–^	4.5 ± 0.3	0.30 ± 0.01	2.5	2.1	218.01	2
3.2	MeO-PCB 2 sulfate	4.09	C_13_H_10_ClSO_5_^–^	1.07 ± 0.09	0.16 ± 0.02	2.6	2.6	233.04, 218.01	2
		4.19		1.8 ± 0.3	0.38 ± 0.03	2.5	2.6	233.04, 218.01, 196.09	2
		4.30		6.6 ± 0.7	1.29 ± 0.09	2.4	2.6	233.04, 218.01	2
3.3	MeO-PCB 2 glucuronide	3.65	C_19_H_18_ClO_8_^–^	0.12 ± 0.02	ND	1.7	ND	233.04, 218.01	2
		3.76		0.45 ± 0.07	ND	1.7	ND	233.04, 218.01	2
3.4	MeO-OH-PCB 2 sulfate	3.76	C_13_H_10_ClSO_6_^–^	0.18 ± 0.04	0.054 ± 0.007	2.2	2.2	249.03, 234.01	2
		3.88		0.07 ± 0.01	0.015 ± 0.001	2.2	2.2	249.03, 234.01	2
4.1	OH-BP sulfate	3.97	C_12_H_9_SO_5_^–^	6.1 ± 0.4	6.1 ± 0.8	2.2	2.3	185.06	2
4.2	MeO-BP sulfate	3.95	C_13_H_11_SO_5_^–^	41 ± 4	6.6 ± 0.4	1.9	2.1	199.08, 184.05, 79.96	2
		4.00		106 ± 10	19 ± 2	1.9	2.0	199.08, 184.05, 79.96	2
4.3	MeO-BP glucuronide	3.51	C_19_H_19_O_8_^–^	2.7 ± 0.7	ND	2.3	ND	199.08, 184.05	2
4.4	MeO-OH-BP sulfate	3.65	C_13_H_11_SO_6_^–^	25 ± 2	2 ± 1	1.7	1.9	215.07, 200.05, 79.96	2
4.5	OH-BP cysteine	3.75	C_15_H_14_NSO_3_^–^	26 ± 3	ND	3.7	ND	201.04, 127.09	2

a HepG2 cells were exposed for 24 h to PCB 2 [high concentration (10 μM) or low concentration (3.6 nM)] as described in the Experimental Section; metabolites were extracted from the cell culture medium by QuEChERS extraction, and extracts were analyzed by LC-Orbitrap MS. Most of these PCB 2 metabolites were also detected in LC-QTof analyses of the same extracts (Table S1). The corresponding MS and MS^2^ spectra are provided in Figures S1–S13.

b Injections for both LC-MS and MS/MS analysis were performed on an LC-Orbitrap MS with an Acquity UPLC BEH C18 column.

c The intensities for PCB 2 metabolites (classes 1–3) are MRI intensities. The intensities for BP metabolites are normalized intensities. For the calculation of MRI or normalized intensities, see the Experimental Section.

d ND, not detected.

e The accurate mass difference in parts per million was calculated as the absolute value of (measured mass – calculated mass)/calculated mass × 10^6^.

f Confidence levels for identifying PCB metabolites were assigned using the Schymanski framework:^96^ level 1, metabolites identified on the basis of accurate mass, isotope pattern, MS, MS2, and authentic standards; level 2, metabolites identified on the basis of accurate mass, isotope pattern, MS, and MS/MS; level 3, metabolites identified on the basis of accurate mass, isotope pattern, and MS, but not MS/MS.

#### Identification of Dihydroxylated PCB 2 Metabolites and Their Conjugates

Two OH-PCB 2 sulfate isomers (class 2.1) were detected in incubations with PCB 2. Both metabolites are likely sulfated 4,5- or 3′,4′-substituted PCB 2 catechols. These substitution patterns are proposed on the basis of our studies demonstrating that PCBs are metabolized to *ortho*,*para*-disubstituted metabolites in HepG2 cells.^29,30^ The OH-PCB 2 sulfates detected herein can be formed by sulfation of the corresponding dihydroxylated PCB 2 or, similar to rats,^70^ by the oxidation of PCB 2 sulfates. No dihydroxylated PCB 2 metabolites or the corresponding OH-PCB 2 glucuronides were detected.

*In vitro* metabolic studies demonstrate that OH-PCBs are readily oxidized to dihydroxylated PCB metabolites.^54,55,72,73^ In the case of PCB 3, three dihydroxylated PCB isomers are formed by rat liver microsomes.^23^ A dihydroxylated PCB 3 metabolite was a key PCB 3 metabolite formed by HepG2 cells.^30^ Dihydroxylated PCB 2 metabolites were not detected in this study, probably due to their rapid biotransformation to other metabolites, including the formation of OH-PCB 2 sulfates and, as shown *in vitro*, reactive PCB 2 quinones.^74,75^

#### Identification of Methoxylated (MeO) PCB 2 Metabolites and Their Conjugates

**Figure 1:**
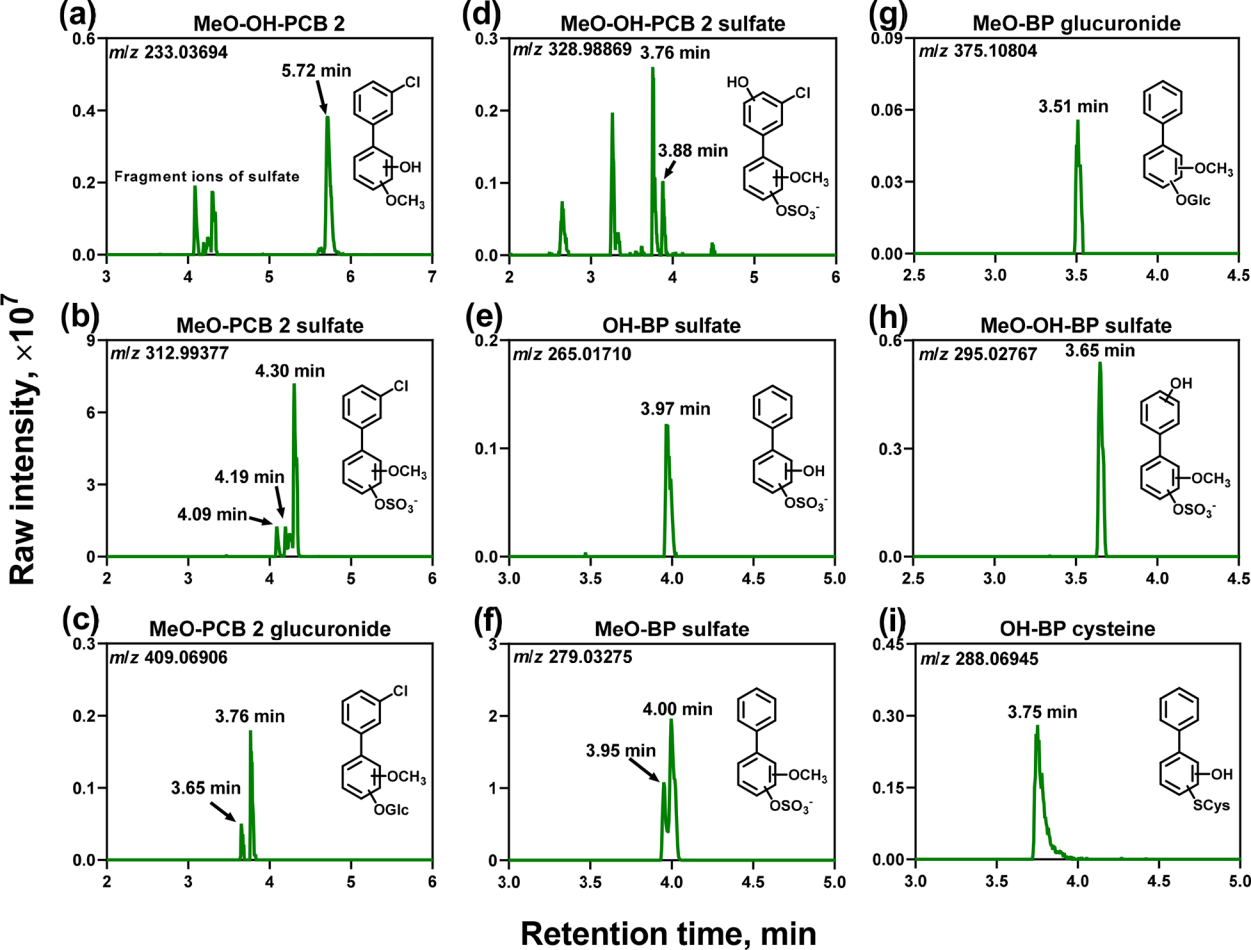
Metabolism of PCB 2 by HepG2 cells results in complex PCB metabolite mixtures. Representative metabolites include methoxylated metabolites [(a) MeO-OH-PCB 2 (*m*/*z* 233.03694), (b) MeO-PCB 2 sulfate (*m*/*z* 312.99377), (c) MeO-PCB 2 glucuronide (*m*/*z* 409.06906), and (d) MeO-OH-PCB 2 sulfate (*m*/*z* 328.98869)] and potential dechlorinated metabolites [(e) OH-BP sulfate (*m*/*z* 265.01710), (f) MeO-BP sulfate (*m*/*z* 279.03275), (g) MeO-BP glucuronide (*m*/*z* 375.10804), (h) MeO-OH-BP sulfate (*m*/*z* 295.02767), and (i) OH-BP cysteine (*m*/*z* 288.06945)]. For each metabolite class, up to three isomers were observed (Table 1). LC-Orbitrap MS analyses were performed in the negative mode. The extracted ion chromatograms are based on the calculated accurate masses of each metabolite class, with a mass window of 10 ppm. They are presented for samples from the high-PCB 2 concentration group. For the relative levels of PCB 2 or BP metabolites between the high- and low-PCB 2 concentration groups, see Table 1. For selected MS and MS/MS spectra, see Figures S1–S13. ND, not detected.

Four classes of novel methoxylated PCB 2 metabolites were detected in cell culture medium from PCB 2-exposed HepG2 cells, including one MeO-OH-PCB 2 [class 3.1 (Figure 1a)], three MeO-PCB 2 sulfates [class 3.2 (Figure 1b)], two MeO-PCB 2 glucuronides [class 3.3 (Figure 1c)], and two MeO-OH-PCB 2 sulfates [class 3.4 (Figure 1d)]. Disposition studies in several animal species report the formation of MeO-OH-PCB (class 3.1) metabolites.^34,76,77^ However, the corresponding MeO-PCB sulfate and glucuronide conjugates (classes 3.2–3.4) were not observed in these animal studies. The formation of all class 3 metabolites is consistent with the metabolism of PCB 11 in HepG2 cells.^29^ Analogous class 3 metabolites, but not class 3.1 metabolites, were also detected in HepG2 cells exposed to *para*-chlorinated PCB 3.^30^ These methoxylated PCB metabolites are likely formed by the methylation of PCB catechol metabolites by catechol-*O*-methyltransferase (COMT).^78,79^ Human biomonitoring studies are needed to confirm the formation of MeO-PCB metabolites in humans.

#### Identification of BP Metabolites and Their Conjugates

An intriguing observation is the presence of BP metabolites (class 4) in the cell culture medium from PCB 2-exposed HepG2 cells, including one OH-BP sulfate [class 4.1 (Figure 1e)], two MeO-BP sulfates [class 4.2, two isomers (Figure 1f)], one MeO-BP glucuronide [class 4.3 (Figure 1g)], one MeO-OH-BP sulfate [class 4.4 (Figure 1h)], and one OH-BP cysteine [class 4.5 (Figure 1i)]. Because no experimental fragmentation patterns were available for the OH-BP cysteine metabolite (class 4.5), we confirmed the presence of this metabolite with an *in silico*-predicted MS/MS spectrum (Figure S13). The BP metabolites were not detected in control cells incubated with DMSO only (Figure S15). Glc, glucuronide.

### Evidence Supporting the Formation of Dechlorinated Metabolites in HepG2 Cells

The BP metabolites observed in the Nt-HRMS analysis represent an unexpected observation. Similarly, we observed dechlorinated metabolites (i.e., monochlorinated PCB metabolites) in our PCB 11 metabolic study (Figures S16–S20).^29^ In the earlier study, we attributed the formation of monochlorinated PCB metabolites to a small PCB 2 impurity in the PCB 11 sample used in the earlier study. The authentication of the PCB 2 batch used herein revealed a small BP impurity (775 ng of BP/mg of PCB 2) that may explain the trace amounts of BP metabolites. In contrast, BP metabolites were not detected in our metabolic study with PCB 3.^30^ Importantly, the PCB 3 batch used in the earlier study contained 6 times higher levels of BPs (i.e., 4.0 μg of BP/mg) compared to that of the PCB 2 sample used in this study. The following evidence indicates that the BP metabolites are formed by the dechlorination of PCB 2 metabolites in HepG2 cells.

### Anticipated Instrument Detection Limits for BP Metabolites

We could not quantify the BP metabolite levels using Nt-HRMS because authentic standards are unavailable. However, the metabolite levels formed from the BP impurity, especially in incubations with low PCB 2 concentration, were likely below the instrument detection limit. This interpretation is supported by our analogous PCB 3 metabolic study, in which we did not detect BP metabolites despite the presence of a BP impurity in PCB 3.^30^ Furthermore, the BP contents in the experiments with high and low PCB 2 concentrations were 28.5 pmol and 10.4 fmol, respectively. However, we detected four BP metabolite peaks with infinite signal:noise (S:N) ratios in samples from PCB 2 exposure cells with a low concentration using LC-Orbitrap MS (Table 1). Assuming that BP is completely biotransformed and analyzed as one metabolite [e.g., MeO-BP sulfate (class 4.2)] with 100% recovery, the on-column mass of this major metabolite is 22 fg. This estimate is more than an order of magnitude lower than our instrument’s certified detection limit (e.g., 500 fg for buspirone in full scan mode with an S:N ratio of 100:1).^80^

Consistent with the LC-Orbitrap MS analyses, our less sensitive LC-QTof MS method detected the major BP metabolite class [i.e., MeO-BP sulfate (class 4.2)] with an S:N ratio of 10 in incubations with low PCB 2 concentrations (Table S1). Assuming that the BP impurity was completely biotransformed into the MeO-BP sulfate, this metabolite concentration was estimated to be 0.015 ng/mL. This value is more than an order of magnitude lower than our instrument detection limit of 5–9 ng/mL for mono- to tetrachlorinated PCB sulfates (Table S4). Despite the differences in the molecular responses, the prediction of the ionization efficiencies suggested that methoxylation only marginally increases the molecular responses of PCB sulfates and, thus, cannot explain the robust signal of these metabolites in the Nt-HRMS analyses.

### Comparison of PCB 2 and PCB 11 Metabolism by HepG2 Cells

**Figure 2:**
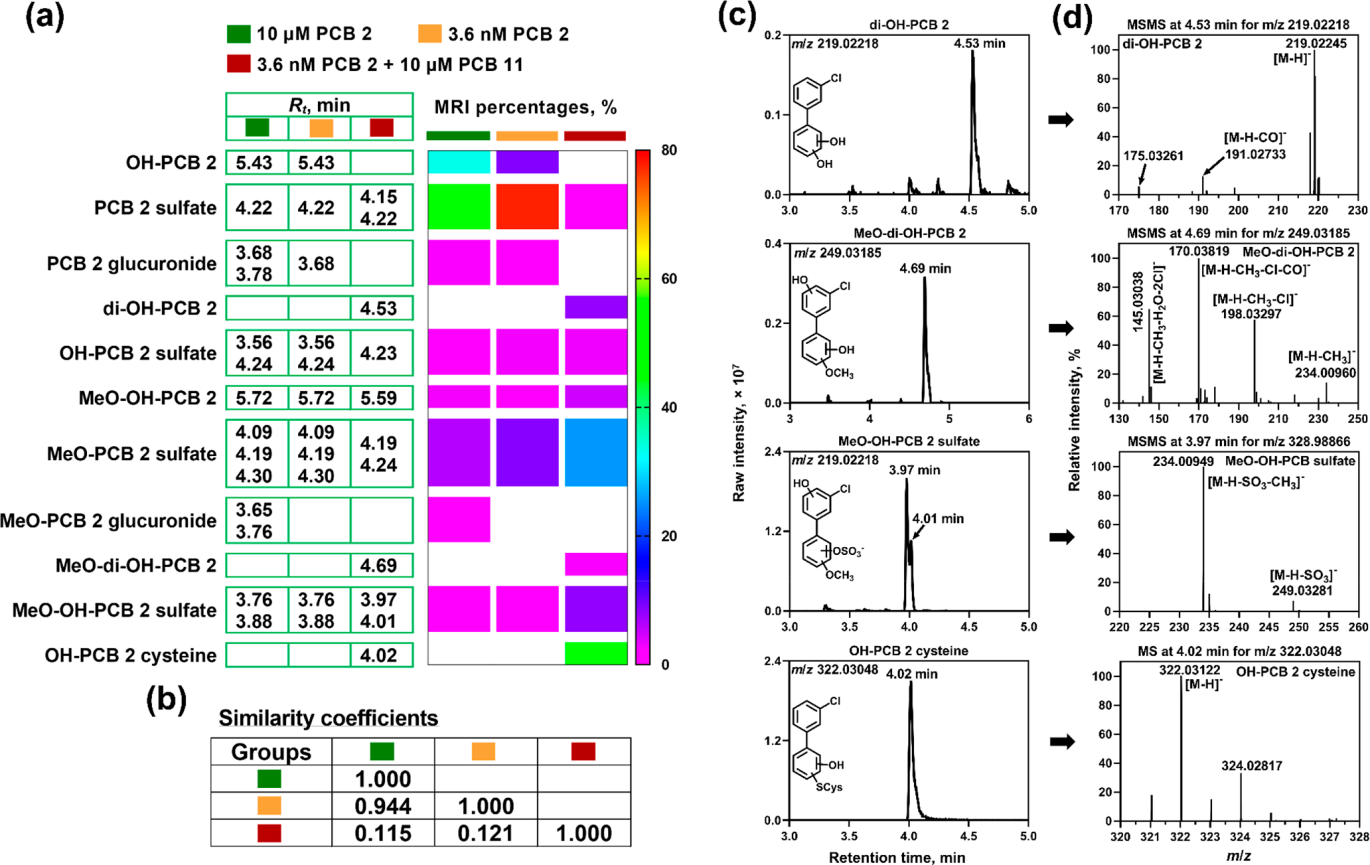
Differences in (a) retention times and the relative metabolite levels [expressed as molecular response-independent (MRI) percentages] and (b) cos θ similarity coefficients of the metabolites suggest the formation of different monochlorinated PCB metabolites in HepG2 cells exposed to PCB 2 (high and low concentrations) or PCB 11 containing a PCB 2 impurity. The retention times are corrected for the internal standard, 3-F,4′-PCB 3 sulfate, to account for batch-to-batch variability in the retention times. The analyses were performed in negative mode on an LC-Orbitrap MS (see the Experimental Section for additional details). (c) The extracted ion chromatograms with a mass window of 10 ppm and (d) the MS or MS/MS data confirm the formation of putative PCB 2 metabolites in incubations of HepG2 cells with PCB 11 containing a PCB 2 impurity. The studies with the PCB 2-contaminated PCB 11 have been described previously.^29^ For the MS and MS/MS spectra of other PCB 2 metabolites from PCB 2-contaminated PCB 11 incubations listed in Table S5, see Figures S16–S20.

Complex mixtures of monochlorinated PCB metabolites were detected in our PCB 11 metabolic study in HepG2 cells (Table S5).^29^ A comparison of the metabolite mixture from this earlier study and the PCB 2 metabolite mixture formed herein suggests that HepG2 cells form dechlorinated metabolites from PCB 11. The differences in the monochlorinated PCB metabolite profile between the PCB 11 and PCB 2 experiments, revealed by a quantitative heat map with MRI intensities (Figure 2a) and cos θ similarity coefficients (Figure 2b), suggest that the monochlorinated PCB metabolites formed in the PCB 11 experiment are derived from PCB 11 and not a PCB 2 impurity. First, four classes of monochlorinated PCB metabolites were formed in the incubations with PCB 11 but not PCB 2, including di-OH-PCB, MeO-di-OH-PCB, MeO-OH-PCB sulfate, and OH-PCB cysteine metabolites (Figure 2c,d). Moreover, the monochlorinated metabolites formed from PCB 11-exposed cells are mostly disubstituted metabolites, such as OH-PCB 2 cysteine (47 ± 10%) and MeO-PCB 2 sulfate (25 ± 9%). In contrast, monosubstituted metabolites were the major metabolites in HepG2 cells exposed to PCB 2.

Finally, PCB 2 and PCB 11 incubations had four putative dechlorinated metabolite classes in common (Figure 2a). Briefly, OH-BP sulfate, OH-BP cysteine, MeO-BP sulfate, and MeO-OH-BP sulfate were observed in our study with PCB 2 (Table 1). The analogous monochlorinated metabolite classes were detected in the case of PCB 11, including OH-PCB 2 sulfate, OH-PCB 2 cysteine, MeO-PCB 2 sulfate, and MeO-OH-PCB 2 sulfate (Table S5). In contrast, no dechlorinated BP metabolites were observed in our study with PCB 3.^30^ This evidence supports the formation of dechlorinated metabolites from *meta*- but not *para*-chlorinated LC-PCBs by HepG2 cells. *meta*- but not *para*-chlorination likely favors the formation of intermediates involved in the dechlorination reactions for electronic or steric reasons;^31−34^ however, more research is needed to characterize the formation of dechlorinated metabolites by human cytochrome P450 enzymes.

### Metabolism of PCB 2 or Its Metabolites by HLMs

**Figure 3:**
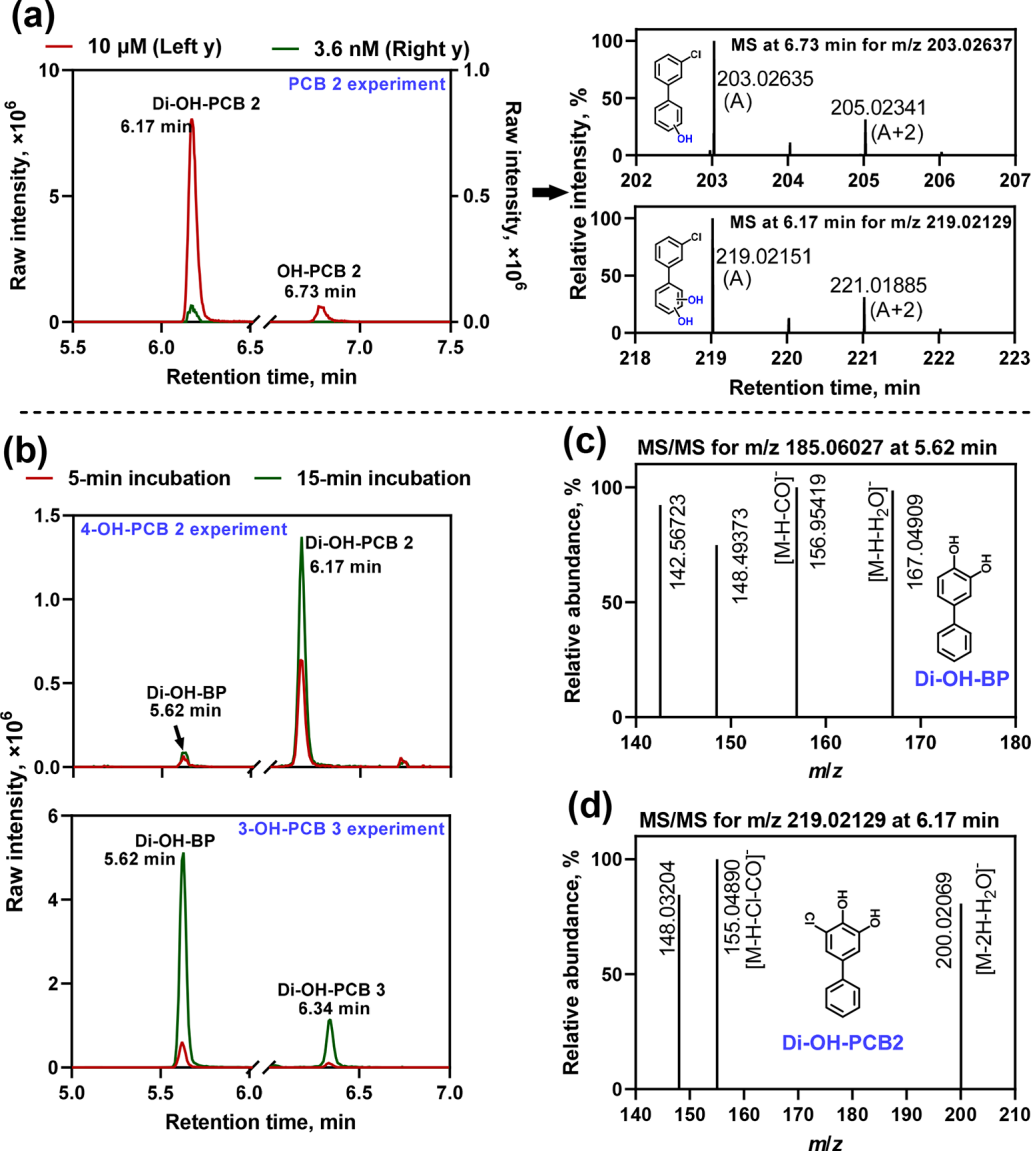
(a) Extracted ion chromatograms (EICs) at *m*/*z* 203.02637 and 219.02129 (with a mass window of 10 ppm) and MS spectra showing the measured accurate masses of the molecular ion and the isotopic pattern of chlorine support the formation of mono- and dihydroxylated PCB 2 metabolites (OH-PCB 2 and di-OH-PCB 2, respectively) in HLM incubations with 3.6 nM or 10 μM PCB 2. For the time course of the formation of these metabolites, see Figure S21. (b) EICs at *m*/*z* 185.06027 and 219.02129 show the presence of a dihydroxylated biphenyl metabolite (di-OH-BP, a dechlorinated metabolite eluting at 5.62 min) and dihydroxylated PCB metabolites (di-OH-PCB 2 eluting at 6.17 min and di-OH-PCB 3 eluting at 6.34 min) in HLM incubations with 10 μM 4-OH-PCB 2 or 3-OH-PCB 3 for both 5 and 15 min. The featured fragment ions measured in the MS/MS spectra support the identification of (c) di-OH-BP (*m*/*z* 185.06027) in the HLM incubation with 10 μM 4-OH-PCB 2 or 3-OH-PCB 3 and (d) di-OH-PCB 2 (*m*/*z* 219.02129, eluting at 6.17 min) in the HLM incubation with 10 μM 4-OH-PCB 2. The MS/MS spectrum of di-OH-PCB 3 was not collected in the data-dependent MS/MS measurement due to its low intensity. The MS spectra showing the accurate mass of the molecular ions and the isotopic pattern of chlorine of the di-OH-PCB metabolites identified in the HLM incubations with 4-OH-PCB 2 or 3-OH-PCB 3 are provided in Figure S22. For more information regarding the metabolites identified in HLM incubations, see Table S6.

Metabolic studies with HLM were performed to assess whether dechlorinated metabolites formed from PCB 2 or its oxidative metabolites. We detected one monohydroxylated and one dihydroxylated PCB 2 metabolite in the HLM incubations (Figure 3a). The monohydroxylated metabolite was not detected at the lower PCB 2 concentration. The levels of both metabolites increased with incubation time (Figure S21). The monohydroxylated PCB 2 metabolite is likely 4-OH-PCB 2. Alternatively, this metabolite may be hydroxylated at the other *para* position on the nonchlorinated benzene ring, as observed with rat liver P450 enzymes or microsomes.^22,23^ The dihydroxylated metabolite is likely 4,5-diOH-PCB 2 because it coeluted with the dihydroxylated product detected in analogous incubations with 4-OH-PCB 2 (see below). A structurally similar PCB catechol metabolite was observed in metabolic studies with PCB 11, another *meta*-chlorinated PCB congener, in HepG2 cells.^29^ Alternatively, the catechol structure may be formed at positions 3′ and 4′, as observed in the PCB 3 metabolic study with rat liver microsomes.^23^

Dihydroxylated PCB metabolites were detected in HLM incubations with 4-OH-PCB 2 or 3-OH-PCB 3 (Figure 3b). PCB 2 and 4-OH-PCB 2 likely formed the same dihydroxylated metabolite in the HLM incubations on the basis of the retention time of this metabolite. A different dihydroxylated PCB metabolite was formed from 3-OH-PCB 3. Notably, HLM incubations with PCB 2 did not form dechlorinated metabolites; however, the same dihydroxylated dechlorinated biphenyl metabolite was formed from 4-OH-PCB 2 and 3-OH-PCB 3 in HLM incubations. This metabolite is produced by replacing chlorine with a hydroxyl group, which results in the formation of the same dechlorinated product (i.e., biphenyl-3,4-diol), as indicated by the retention time (Figure 3b). The identification of both dihydroxylated metabolites was based on the experimentally measured accurate mass of the molecular ion in the MS spectra (Figure S22) and featured fragment ions in the MS/MS spectra, for example, [M – H – H_2_O]^−^, [M – H – CO]^−^, or [M – H – CO – Cl]^−^ (Figure 3c,d), consistent with published catechol fragmentation patterns.^29,30^ We did not detect any sulfate or glucuronide PCB metabolites because the HLM incubations do not contain the respective drug-metabolizing enzymes (i.e., cytosolic SULTs) or cofactors (e.g., uridine diphosphate glucuronic acid, the cofactor of uridine 5′-diphospho-glucuronosyltransferases).

### Metabolic Pathway Leading to Dechlorinated LC-PCB Metabolites in HepG2 Cells

The formation of dechlorinated PCB metabolites has not been reported for human-relevant models. However, disposition studies in rats^31,33^ and rabbits^32,34^ reported the formation of dechlorinated PCB metabolites. Dechlorination was also observed in rats exposed to OH-PCB congeners with the OH group in the *ortho* position to a chlorine substituent.^31^ On the basis of *in vivo* studies, the loss of chlorine from PCBs is thought to occur via an arene oxide intermediate^32−34^ or by direct reductive dechlorination.^31^ The formation of the corresponding sulfate, glucuronide, and cysteine conjugates of the dechlorinated metabolite has not been reported previously.

**Figure 4:**
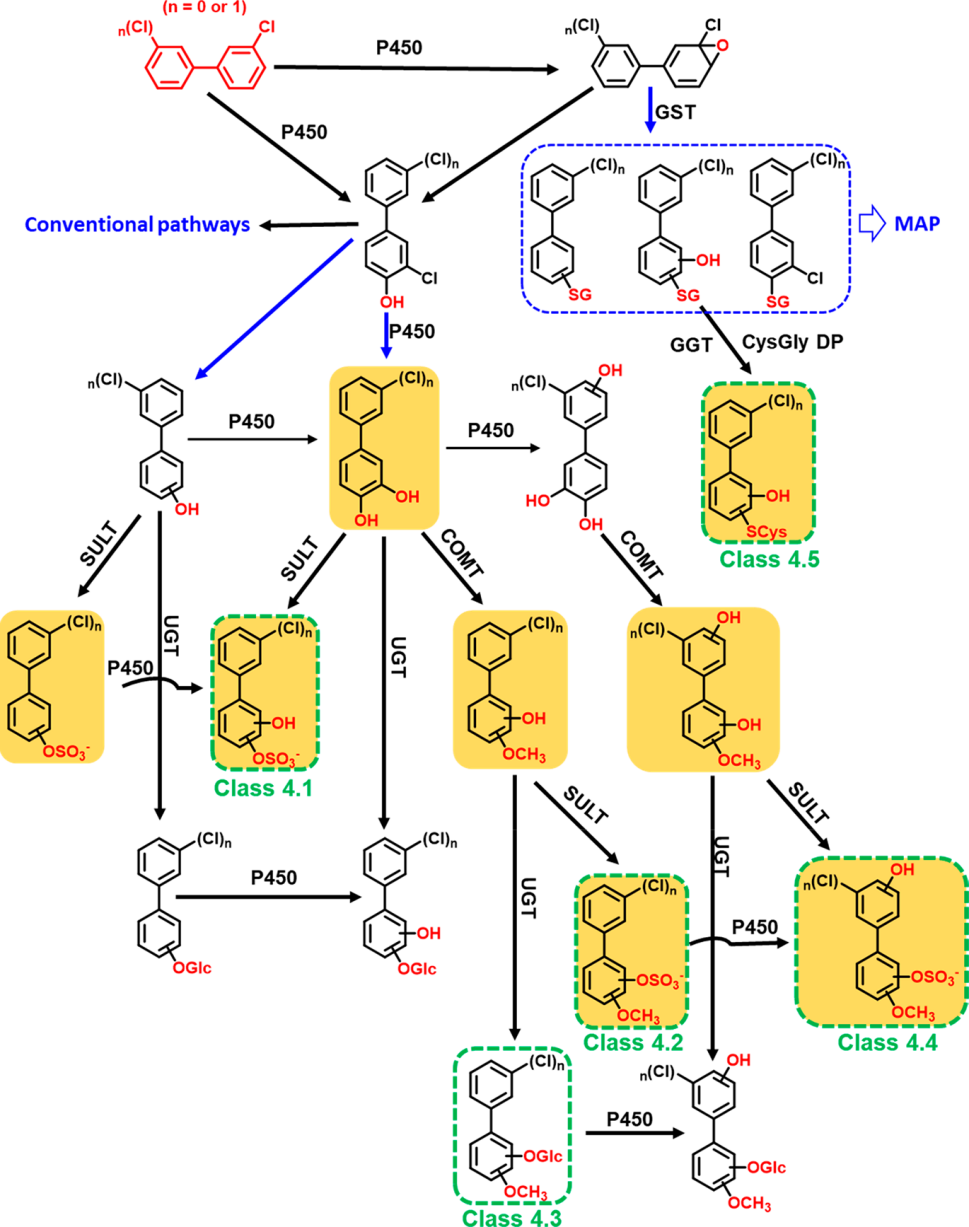
Proposed metabolic pathway showing the potential dechlorination of PCB 2 or, on the basis of our earlier work,^29^ PCB 11 in HepG2 cells. Dechlorination (blue arrow) occurs either via a 3,4-arene oxide or by reductive dechlorination of hydroxylated PCB metabolites with the hydroxyl group *ortho* to the chlorine substituent. Structures shown in green boxes were detected in this metabolic study with PCB 2. Monochlorinated metabolites on an orange background were detected in our earlier metabolic study with PCB 11^29^ but were not identified as PCB 11 metabolites due to the limited experimental evidence. The placement of the functional groups is for the purpose of illustration only and does not indicate their actual position. The classical metabolic scheme (conventional pathways) of PCB 2 is depicted in Figure S23. Abbreviations: P450, cytochrome P450 enzyme; Glc, glucuronide; DHDH, dihydrodiol dehydrogenase; SULT, sulfotransferase; EH, epoxide hydrolase; UGT, uridine 5′-diphospho-glucuronosyltransferase; COMT, catechol-*O*-methyltransferase; GST, glutathione *S*-transferase; SG, glutathione; SCys, cysteine; GGT, γ-glutamyl transpeptidase; CysGly DP, cysteinylglycine dipeptidase; MAP, mercapturic acid pathway.

On the basis of the available evidence, we propose a metabolic pathway leading to dechlorinated PCB 2 or PCB 11 metabolites (Figure 4). Briefly, we suggest that dechlorinated hydroxylated metabolites of PCB 2 or PCB 11 are formed via a 3,4-arene oxide intermediate, followed by the formation of monohydroxylated metabolites with a hydroxyl group *ortho* to a chlorine atom, for example, 4-OH-PCB 2 or 4-OH-PCB 11 (3,3′-dichlorobiphenyl-4-ol). Next, these monohydroxylated PCB metabolites undergo replacement of the chlorine substituent ortho to the hydroxyl group to form dihydroxylated dechlorinated metabolites (e.g., di-OH-BP metabolites derived from PCB 2 in HLM incubations or di-OH-PCB 2 metabolites derived from PCB 11 in HepG2 cells in culture). In addition, 4-OH-PCB 2 or 4-OH-PCB 11, formed by oxidation of the corresponding parent PCB, can undergo reductive dechlorination to form OH-BP or OH-PCB 2, respectively. We did not detect these transient OH-BP and di-OH-BP metabolites in incubations of HepG2 cells with PCB 2 or PCB 11; however, the additional PCB 2 sulfate (Table S5) and di-OH-PCB 2 isomers detected in studies with PCB 11 (Figure 2) were potentially formed through this pathway. Subsequently, the other dechlorinated metabolites detected in this work and our earlier study^29^ are formed via classical LC-PCB metabolic pathways (Figure S23).

The addition of glutathione to a PCB arene oxide intermediate can result in OH-BP (or OH-PCB 2) glutathione adducts with dechlorination or PCB 2 (or PCB 11) glutathione adducts with the loss of water but without dechlorination. We did not detect any PCB glutathione metabolites or metabolites of the mercapturic acid pathway in this work or our earlier study with PCB 11.^29^ However, we observed dechlorinated cysteine metabolites (class 4.5) in both studies (Figure 2, Table 1, and Table S5). The cysteine adducts may be formed from the corresponding glutathione adducts through a stepwise degradation catalyzed by γ-glutamyl transpeptidase and cysteinyl-glycine dipeptidase.^21^ Similarly, metabolic studies with 1-chloro-2,4-dinitrobenzene demonstrate that HepG2 cells rapidly metabolize this chlorobenzene to cysteine S-conjugates.^81^

### Toxicological Implications: Formation of Toxic PCB 2 Metabolites

The metabolites of PCB 2 are potentially associated with toxic outcomes in humans. For example, structurally similar OH-PCB metabolites interact with diverse cellular targets implicated in adverse health effects, such as aryl hydrocarbon and estrogen receptors.^82,83^ Other lower chlorinated OH-PCBs can act as endocrine-disrupting chemicals via the estrogen receptor or by inhibiting estrogen sulfotransferase.^84−86^ OH-PCBs and PCB sulfates can also disrupt thyroid homeostasis by acting as high-affinity ligands of transthyretin.^50^ Importantly, the formation of BP metabolites may be associated with adverse effects linked to BP exposure, such as genotoxicity and carcinogenesis.^87^

Moreover, PCB 2 biotransformation results in reactive or redox-active intermediates, such as arene oxides, catechols, hydroquinones, and quinones, of PCB 2 and BP.^21,74,88^ Both arene oxides and quinones can readily react with cellular nucleophiles, including proteins and DNA. Genotoxic effects have been reported for the reactive BP metabolites formed in incubations with PCB 2.^89,90^ Furthermore, catechols, hydroquinones, and quinones can redox cycle, thus altering the cellular redox homeostasis.^38,39^ For example, PCB 2 undergoes bioactivation to redox-active di- and trihydroxylated metabolites with a catechol substitution pattern (Figure S23). However, these metabolites are readily converted to hydroxylated or methoxylated sulfate and glucuronide conjugates. These downstream metabolites are not redox-active, cannot be oxidized to toxic PCB quinones, and, in the case of sulfate and glucuronide conjugates, are readily eliminated with urine or feces.^21,91^ Because the chemical structure of the reactive metabolites tentatively identified in this work and other studies is unknown, and because authentic standards are not available, their toxicological relevance remains unknown and warrants further attention. The dechlorinated metabolites may be less persistent in the body because they are more readily metabolized; however, the resulting metabolites may also be more toxic.

### Changes in Endogenous Metabolism in Media from PCB 2-Exposed HepG2 Cells

**Figure 5:**
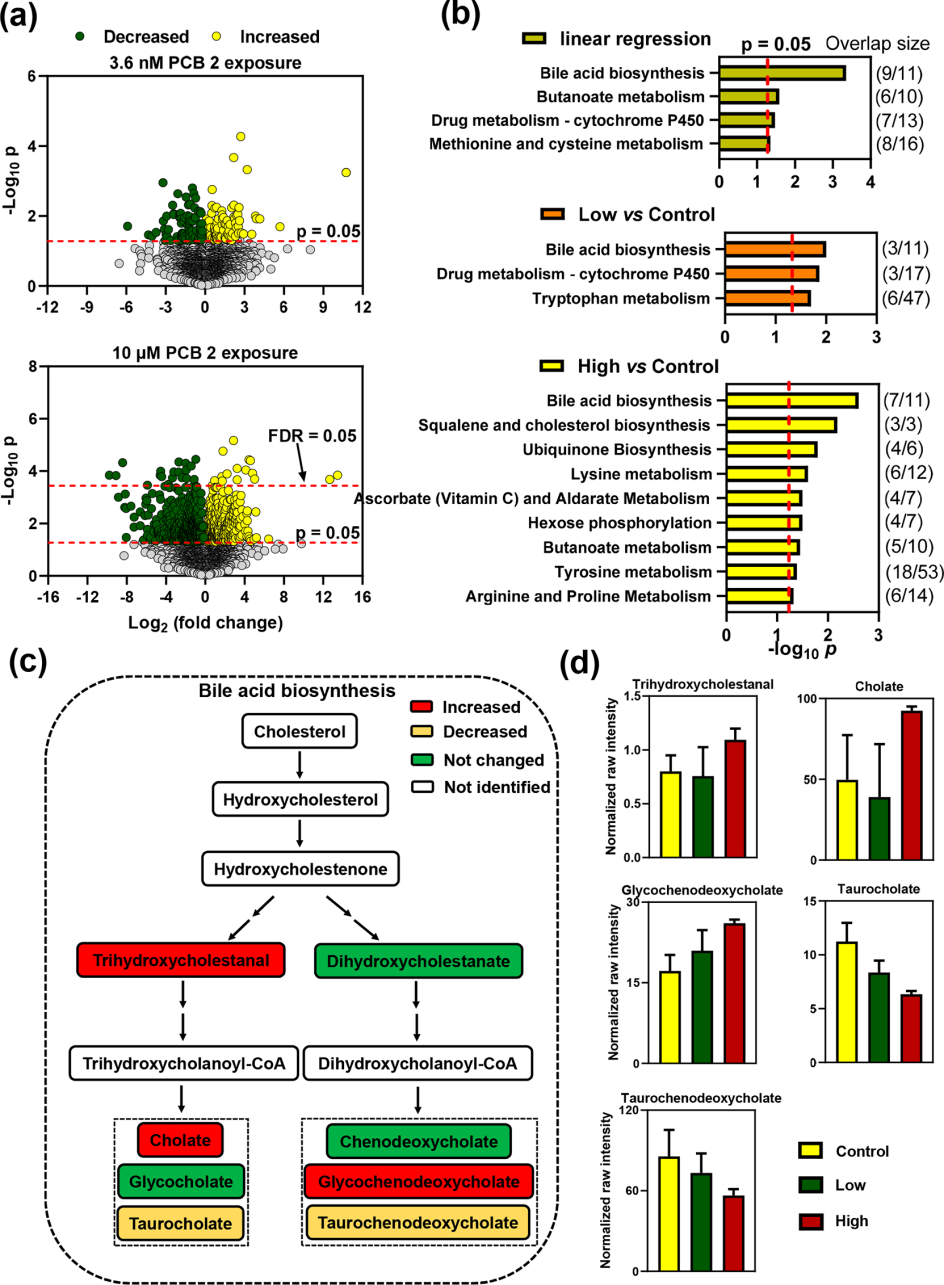
Untargeted metabolomic analysis of medium samples revealed concentration-dependent effects of PCB 2 on the metabolome in HepG2 cells exposed to 3.63 nM PCB 2, 10 μM PCB 2, or vehicle (DMSO). (a) Univariate statistical analyses identified 170 and 787 metabolic features above a *p* = 0.05 threshold when comparing low and high PCB 2 exposures, respectively, to controls. In this analysis, 32 features were above the FDR = 0.05 threshold when comparing high PCB 2 exposures and control groups. (b) Pathway enrichment analyses using feature lists with raw *p* values from the linear regression with concentration and univariate statistical analyses of low PCB 2 vs control and high PCB 2 vs control identified four, three, and 12 significantly affected pathways, respectively (*p* < 0.05). Bile acid biosynthesis was the top enriched pathway in all three pathway enrichment analyses. The number of features altered by PCB 2 in these pathways is depicted as overlap/total features. (c) Several metabolites in the bile acid biosynthesis pathway were significantly affected by PCB 2 exposure. (d) Levels of these metabolites, plotted as normalized raw intensities, increased for trihydroxycholestanal (*p* = 0.0373), cholate (*p* = 0.0232), and glycochenodeoxycholate (*p* = 0.0165) and decreased for taurocholate (*p* = 0.0262) and taurochenodeoxycholate (*p* = 0.0601). The accurate *m*/*z* values, retention times, adducts, significances, and confidence scores of the metabolite annotations in the bile acid biosynthesis pathway are listed in Table S7.

The diverse PCB 2 metabolites formed by HepG2 cells affected the endogenous metabolome. Briefly, untargeted metabolomic analyses of the medium revealed significant changes in features between incubations with PCB 2 and vehicle (DMSO). Univariate analysis identified 170 and 787 of 5405 features that were significantly changed (*p* < 0.05) between controls and the low and high PCB 2 concentrations, respectively (Figure 5a). Exposure to the high PCB 2 concentration had broader effects on the metabolome than exposure to the low PCB 2 concentration. After adjustment for the FDR, 32 features were significantly different between high-PCB 2 exposure and control experiments. Further statistical analysis of concentration-dependent changes using a linear regression model identified 966 significant features with a *p* of <0.05 and 203 features with an FDR of <0.05.

Subsequently, pathway enrichment analyses identified four, three, and 12 significantly affected pathways for linear regression with concentration, low PCB 2 versus control, and high PCB 2 versus control, respectively (Figure 5b). The bile acid biosynthesis was the top enriched pathway in all three pathway enrichment analyses. Also, amino acid metabolic pathways (i.e., lysine and tyrosine metabolism) were significantly altered following PCB 2 exposure. These pathways are also affected by PCB 11 exposure in HepG2 cells.^29^ Thus, altered amino acid metabolism appears to be a common effect of LC-PCBs in HepG2 cells.

The relative levels of several intermediates in the bile acid biosynthesis (Figure 5c) were affected in HepG2 cells exposed to PCB 2. For example, levels of cholate, trihydroxycholestanal, and glycochenodeoxycholate increased and levels of taurocholate and taurochenodeoxycholate decreased following PCB 2 exposure (Figure 5d). There is evidence that PCBs alter bile acid homeostasis in the literature. PCB exposure lowers hepatic cholesterol 7α-hydroxylase (CYP7A1) gene expression levels in rats,^92^ a key enzyme in the synthesis of bile acids. Moreover, two studies found a positive correlation between PCB exposure and cholesterol levels in rat and human serum.^93,94^ The extent to which the findings from this *in vitro* study reflect the effects of PCB exposure *in vivo* is unclear because emerging evidence demonstrates that the gut microbiome plays a role in modulating PCB–bile acid interactions.^95^ Therefore, further studies are needed to investigate how PCB 2 exposure affects this pathway and other metabolic pathways in humans and if these effects are associated with toxic outcomes.

## Supporting Information Available

Experimental details regarding biphenyl analysis in PCB samples, cell culture experiments, metabolite extraction and analysis, ADMET Predictor and MetaDrug prediction results, MS data for the identification of PCB 2 and PCB 11 metabolites, limits of detection of authentic standards, metabolic data from the metabolomics analysis, estimated ionization efficiencies of PCB 2 metabolites, MS and MS/MS data of BP or PCB 2 metabolites in HepG2 and HLM experiments, and classical metabolic pathways of PCBs (PDF)
