# Supplementary material for: Metabolism of 3-Chlorobiphenyl (PCB 2) in a Human-Relevant Cell Line: Evidence of Dechlorinated Metabolites
Source: Environ Sci Technol. 2022 Aug 22;56(17):12460–72. doi: 10.1021/acs.est.2c03687 (PMC9573771; doi:10.1021/acs.est.2c03687)
Supplement: Supplementary file 1 — es2c03687_si_001.pdf [file es2c03687_si_001.pdf]

SUPPORTING INFORMATION

METABOLISM OF 3-CHLOROBIPHENYL (PCB 2) IN A HUMAN-RELEVANT CELL LINE: EVIDENCE OF DECHLORINATED METABOLITES

Chun-Yun Zhang[1,2], Xueshu Li,[2] Susanne Flor[2], Patricia Ruiz[3], Anneli Kruve[4], Gabriele Ludewig[2], Hans-Joachim Lehmler[2,*]

[1]Hubei Key Laboratory of Regional Development and Environmental Response, Faculty of Resources and Environmental Science, Hubei University, Wuhan 430062, China. [2]Department of Occupational and Environmental Health, The University of Iowa, Iowa City, Iowa 52242, United States. [3]Office of Innovation and Analytics, Simulation Science Section, Agency for Toxic Substances and Disease Registry, Atlanta, Georgia 30333, United States. [4]Department of Materials and Environmental Chemistry, Stockholm University, Svante Arrhenius Väg 16, 10691, Stockholm, Sweden.

Corresponding Author:
Dr. Hans-Joachim Lehmler
The University of Iowa
Department of Occupational and Environmental Health
University of Iowa Research Park, #164 MTF
Iowa City, IA 52242-5000
Phone: (319) 335-4981
Fax: (319) 335-4290
e-mail: hans-joachim-lehmler@uiowa.edu

Number of pages: 52
Number of tables: 7
Number of figures: 23

Table of Contents

Quantification of the biphenyl (BP) impurity in PCB 2	S5
Cell culture	S6
Extraction of PCB metabolites from the cell culture medium	S6
Extraction of PCB metabolites from human liver microsomal incubation	S6
Ultra-Performance Liquid Chromatography-Quadrupole Time-of-flight Mass Spectrometric (LC-QTof MS) analysis	S7
Ultra-Performance Liquid Chromatography-Orbitrap Mass Spectrometric (LC-Orbitrap MS) analysis	S8
In silico predictions of human-relevant PCB 2 metabolites	S9
Metabolite identification and quality assurance/quality control	S12
**Table S1.** Summary of PCB 2 metabolites identified in cell culture medium from HepG2 cells exposed to 10 µM or 3.6 nM PCB 2 for 24 h analyzed by LC-QTof MS	S13
**Table S2.** Metabolism of PCB 2 and its metabolites by human cytochrome P450 enzymes and UDP-glucuronosyltransferases (UGTs) predicted by ADMET Predictor	S14
**Table S3.** Metabolites formed from PCB 2 and its metabolites in humans, as predicted by MetaDrug	S17
**Table S4.** The limits of detection (LODs) of mono- to tetra-chlorinated PCB sulfates analyzed by LC-QTof MS	S22
**Table S5.** Several likely PCB 2 metabolite classes (i.e., monochlorinated PCB metabolites) were detected by LC-Orbitrap MS analysis in HepG2 cell culture medium exposed to 10 µM PCB 11	S23
**Table S6.** Summary of BP or PCB 2 metabolites identified in HLM incubation with 10 µM PCB 2, 4-OH-PCB 2, or 3-OH-PCB 3 for 15 min	S24
**Table S7.** The *m/z*, retention times, p-values, and confidence levels of the metabolites in the bile acid biosynthesis pathway identified through the metabolomic analysis for HepG2 cells exposed to high and low concentrations of PCB 2 or vehicle for 24 h	S25
**Fig. S1.** OH-PCB 2 was present in the cell culture medium from HepG2 cells exposed to 10 µM PCB 2	S26
**Fig. S2.** PCB 2 sulfate was present in the cell culture medium from HepG2 cells exposed to 10 µM PCB	S27
**Fig. S3.** Two PCB 2 glucuronide isomers were present in the cell culture medium from HepG2 cells exposed to 10 µM PCB 2	S28
**Fig. S4.** Two OH-PCB 2 sulfates were present in the cell culture medium from HepG2 cells exposed to 10 µM PCB 2	S29
**Fig. S5.** One MeO-OH-PCB 2 metabolite (eluting at 5.72 min) was present in the cell culture medium from HepG2 cells exposed to 10 µM PCB 2	S30

Fig. S6. Three MeO-PCB 2 sulfate isomers (eluting at 4.09, 4.19, and 4.30 min) were present in the cell culture medium from HepG2 cells exposed to 10 µM PCB 2 S31

Fig. S7. Two MeO-PCB 2 glucuronide isomers (eluting at 3.65 and 3.76 min) were present in the cell culture medium from HepG2 cells exposed to 10 µM PCB 2 S32

Fig. S8. Two MeO-OH-PCB 2 sulfate isomers (eluting at 3.76 and 3.88 min) were present in the cell culture medium from HepG2 cells exposed to 10 µM PCB 2 S33

Fig. S9. One OH-BP sulfate metabolite (eluting at 3.97 min) was present in the cell culture medium from HepG2 cells exposed to 10 µM PCB 2 S34

Fig. S10. One MeO-BP sulfate isomer (eluting at 3.95 and 4.00 min) was present in the cell culture medium from HepG2 cells exposed to 10 µM PCB 2 S35

Fig. S11. One MeO-BP glucuronide metabolite (eluting at 3.51 min) was present in the cell culture medium from HepG2 cells exposed to 10 µM PCB 2 S36

Fig. S12. One MeO-OH-BP sulfate metabolite (eluting at 3.65 min) was present in the cell culture medium from HepG2 cells exposed to 10 µM PCB 2 S37

Fig. S13. The agreement of the measured and predicted MS/MS spectra of OH-BP cysteine supports its detection in the cell culture medium from HepG2 cells exposed to 10 µM PCB 2 S38

Fig. S14. The logarithmic ionization efficiency values (log*IE*) of eleven classes of PCB 2 metabolites listed in Table 1 and Table S4 were predicted with random forest regression from PaDEL descriptors S39

Fig. S15. The formation of BP metabolites from PCB 2 was supported by the clean background levels in the DMSO controls S40

Fig. S16. Two likely PCB 2 sulfate isomers were present in the cell culture medium from HepG2 cells exposed to 10 µM PCB 11 S41

Fig. S17. A putative OH-PCB 2 sulfate was present in the cell culture medium from HepG2 cells exposed to 10 µM PCB 11 S42

Fig. S18. A putative MeO-OH-PCB 2 metabolite was present in the cell culture medium from HepG2 cells exposed to 10 µM PCB 11 S43

Fig. S19. Two putative MeO-PCB 2 sulfate isomers were present in the cell culture medium from HepG2 cells exposed to 10 µM PCB 11 S44

Fig. S20. A putative MeO-OH-PCB 2 sulfate metabolite (eluting at 4.01 min) was present in the cell culture medium from HepG2 cells exposed to 10 µM PCB 11 S45

Fig. S21. The time course of the formation of (a) OH-PCB 2 and (b) Di-OH-BP in the HLM incubation with 10 µM PCB 2 S46

Fig. S22. The identification of di-OH-PCB metabolites was supported by MS spectra showing the accurate mass of the molecular ion and their isotope patterns for metabolites eluting at (a) 6.17 min and (b) 6.34 min in the HLM incubations with 10 µM 4-OH-PCB 2 and 3-OH-PCB 3, respectively S47

Fig. S23. Metabolism scheme showing the complex metabolism of PCB 2 in HepG2 cells S48

References S49

Quantification of the biphenyl (BP) impurity in PCB 2. The BP content in PCB 2 was quantified with an external calibration method. Briefly, PCB 2 solutions in hexane (1 mg/mL) were prepared in triplicate and analyzed by gas chromatography-mass spectrometry (GC-MS). Standard solutions of BP in hexane with concentrations of 1, 5, 10, 50, 100, 500, and 1000 ng/mL were also prepared and analyzed in parallel. The BP contents in the 1 mg/mL PCB 2 solutions were quantified with the established standard calibration curve of BP. The calibration curve was linear over the concentration range from 1 to 1000 ng/mL with $R^2 > 0.999$. The limit of detection (LOD) and quantification (LOQ) of BP in neat PCB 2, calculated as 3- and 10-times the ratios of deviation of the intercept (Δy) over the slope (s) of the standard calibration curves (i.e., $LOD = 3 \times \Delta y/s$ and $LOQ = 10 \times \Delta y/s$), are 10 ng/mL and 33 ng/mL, respectively. In a separate experiment, the LOD and LOQ for the determination of BP in neat PCB 3 were 5 ng/mL and 16 ng/mL, respectively.

GC-MS analyses were performed on an Agilent 7890A gas chromatograph equipped with an SLB-5ms capillary column (30 m length, 250 µm inner diameter, 0.25 µm film thickness; Supelco, St Louis, MO, USA) and an Agilent 5975C MS system with a triple-axis detector and electron ionization (EI) source. Measurements were performed in the selected ion monitoring (SIM) mode at *m/z* 154 and 155 for BP with a collision energy of 70 eV. Helium was used as the carrier gas at a 1 mL/min constant flow rate. The GC column temperature program started from 50 ^{o}C, held for 1 min, increased to 170 ^{o}C by 15 ^{o}C/min, increased to 180 ^{o}C by 1 ^{o}C/min, increased to 280 ^{o}C by 15 ^{o}C/min, and held for 5 min. The transfer line temperature was 280 ^{o}C. The MS source and quadrupole temperatures were 230 ^{o}C and 150 ^{o}C, respectively. Based on this analysis, the PCB 2 used in the present study contained 775 ng BP per mg PCB. The same

analytical method was employed to authenticate the PCB 3 batch used in our earlier metabolism study with PCBs.[1] This PCB 3 samples contained 4.9 µg BP per mg PCB (0.4 % w/w).

Cell culture. HepG2 cells were purchased from American Type Culture Collection (ATCC) (Manassas, VA, USA). The authenticity of the human hepatocellular carcinoma cell line HepG2 was confirmed by analyzing genomic DNA conducted by the University of Arizona Genetics Core (Arizona Research laboratories, Tucson, AZ, USA). The HepG2 cells used in this study were between passages 18 through 35. Cells were maintained in complete medium (MEM supplemented with 10 % FBS, 100 U/mL penicillin, 100 µg/mL streptomycin, and 2 mM l-glutamine) in a humidified incubator with 5% CO_2 at 37 °C. The exposure medium contained MEM without FBS but was supplemented with 4.5 mM D-glucose as an energy source, 100 U/mL penicillin, 100 µg/mL streptomycin, and 2 mM l-glutamine. PCB 2 was dissolved in DMSO. The final concentration of DMSO in the medium did not exceed 0.1 % (v/v). This DMSO concentration does not have any effect on cell viability.

Extraction of PCB metabolites from the cell culture medium. The procedure for extracting PCB metabolites from cell culture media samples was published in our earlier study.[2] Briefly, the medium samples (~ 4 mL) were spiked with the internal standards (3-F,4'-OH-PCB 3 and 3-F,4'-PCB 3 sulfate, 200 ng each) and acidified with formic acid (400 µL, 10 % vol/vol in water). Next, magnesium sulfate (1.2 g) and sodium chloride (0.3 g) were added, and samples were extracted two times with acetonitrile (4 mL and 3 mL). Finally, the combined extracts were dried with magnesium sulfate (2 g), and the solvent was exchanged to acetonitrile/water (200 µL; 15:85, vol/vol).

Extraction of PCB metabolites from human liver microsomal incubation. Initially, the quenched incubation samples (about 3.4 mL) were spiked with 3-F,4'-OH-PCB 3 and 3-F,4'-PCB

3 sulfate (100 ng of each), followed by the addition of 4 mL of acetonitrile. Subsequently, a mixture of magnesium sulfate (800 mg) and sodium chloride (200 mg) was added, and the samples were vortexed vigorously, inverted, and centrifuged. Finally, the extracts were passed through hybrid phospholipid solid phase extraction (HybridSPE) cartridges (3 mL, Millipore Sigma, Burlington, Massachusetts, USA) loaded with 3 g of a mixture of anhydrous sodium sulfate and anhydrous magnesium sulfate (1:1, w/w). The cartridges were preequilibrated with 3 mL of acetonitrile before loading the extract. Next, the extracts were evaporated to dryness with a SpeedVac, taken up in 300 µL of acetonitrile, transferred to a clean tube, and the solvent was exchanged to 200 µL of water/acetonitrile (85/15, v/v).

Ultra-Performance Liquid Chromatography-Quadrupole Time-of-flight Mass Spectrometric (LC-QTof MS) analysis. An initial screening for PCB metabolites was performed using an LC-QTof MS. Briefly, the LC-QTof-MS analysis was carried out on a Waters Acquity UPLC (Waters, Milford, MA, USA) coupled with a Waters Q-Tof Premier mass spectrometer. These measurements were performed in the High-Resolution Mass Spectrometry Facility of the University of Iowa (Iowa City, IA, USA). A Waters Acquity BEH C-18 column (2.1 mm inner diameter, 100 mm length, 1.7 µM particle size; Waters) was used for the chromatographic separation of the PCB 2 metabolites with a flow rate of 0.2 mL/min. The mobile phase was (A) water with 0.04 % (v/v) triethylammonium and (B) acetonitrile. The following solvent gradient (% (B)) was used: 0-1 min, 15%; 1-10 min, 15-95 %, 10-15 min, 95 %. Full scans were performed in the ESI^- mode with mass to charge ratios (*m/z*) ranging from 75 to 800 Da at a rate of 0.2 s/scan. Leucine enkephalin was infused (10 µL/min) as the lock mass and analyzed separately in the ESI^- mode. The sampling cone voltage was 35 V. The desolvation gas was operated at 350 ^{o}C with a flow rate of 650 L/h. The capillary voltage was 2.8 kV.

Ultra-Performance Liquid Chromatography-Orbitrap Mass Spectrometric (LC-Orbitrap-MS) analysis. LC-Orbitrap MS analyses were performed at the Center of Mass Spectrometry and Proteomics at the University of Minnesota (Minneapolis, MN, USA) and at the High-Resolution Mass Spectrometry Facility of the University of Iowa (Iowa City, IA, USA) for cell culture and microsomal incubation samples, respectively. The detailed information regarding the conditions of LC-Orbitrap MS analysis is as follows:

Analysis of cell culture media samples. LC-Orbitrap MS analyses of HepG2 cell culture media extracts were carried out with a UPLC (Ultimate 3000 UHPLC+ Focused, Thermo Fisher, Waltham, MA, USA) coupled with a Q Exactive Hybrid Quadrupole-Orbitrap mass spectrometer (LC-Orbitrap MS; Thermo Fisher) at the Center of Mass Spectrometry and Proteomics at the University of Minnesota (Minneapolis, MN, USA) using full scan and MS/MS methods. The PCB2 metabolites were separated with an Acquity UPLC BEH C-18 column (2.1 mm inner diameter, 100 mm length, 1.7 µM particle size; Waters). The flow rate was 0.3 mL/min. The following gradient (% (B) acetonitrile) was used: 0-1 min, 15 %; 1-3 min, 15-60 %; 3-11.5 min, 60-90 %; 11.5-12 min, 90-98 %; 12-12.5 min, 98%; 12.5-13 min 98-15 %; and 13-14 min, 15 %. Water with 10 mM ammonium acetate (pH = 7.0) was used as solvent (A). The Orbitrap MS system was equipped with heated-electrospray ionization (HESI-Ⅱ) probe source and operated in the negative mode using the following conditions: Spray voltage, 3.0 kV; sheath gas flow rate, 50 arb; capillary temperature, 320 °C; auxiliary gas heater, 400 °C; S-lens, 55 V. Full-scan accurate mass spectra were obtained in a scan range from *m/z* 70 to 1050 with a resolution of 70000 FWHM. Targeted MS/MS spectra for PCB 2 metabolites were collected at a collision energy of 30 eV. Since no PCB metabolites were detected in the ESI positive mode in our earlier studies, only ESI negative data were collected and processed.

Analysis of microsomal incubation samples. LC-Orbitrap MS analyses of human liver microsome incubation samples were carried out with a Q-Executive Orbitrap Mass Spectrometer (Thermo Fisher Scientific, Waltham, MA, USA) with a Vanquish Flex ultra-high-performance liquid chromatograph (Thermo Fisher Scientific) with an ACQUITY UPLC-C18 column (particle size: 1.7 µm, 2.1 x 100 mm, Waters, Milford, MA, USA) at the High-Resolution Mass Spectrometry Facility of the University of Iowa. Mobile phase A, 5% acetonitrile in water with 10 mM ammonium formate and 0.1% formic acid, and B, 5% water in acetonitrile with 10 mM ammonium formate and 0.1% formic acid, were used. The mobile phase flow rate was 0.3 mL/min, and the pressure was 4000-8000 psi. The UPLC gradient program started at 5% B, held for 1 min, increased linearly to 95% B, held for 3 min, and returned to 5% B, with a hold for 1 min before the next injection. Solvent blanks (water/acetonitrile, 85/15, v/v) were used to monitor carryover. MS/MS spectra for PCB 2 metabolites were collected at a collision energy of 45 eV.

***In silico* predictions of human-relevant PCB 2 metabolites.** Human PCB 2 metabolites were predicted with ADMET Predictor (Simulations Plus, Lancaster, CA, USA) and MetaDrug (Thompson Reuters, New York, NY, USA), see Tables S2 and S3. These predictions were used in conjunction with published metabolism studies[3, 4] to develop a candidate screening list of possible human PCB 2 metabolites formed by HepG2 cells, as described.[2, 5] ADMET Predictor was also used to identify cytochrome P450 isoforms possibly contributing to the biotransformation of PCB 2. The metabolic pathway proposed by both programs is discussed in the following sections.

Prediction of the biotransformation of PCB 2 with ADMET Predictor. ADMET Predictor suggested the formation of two *para*-hydroxylated PCB 2 metabolites, 4-OH-PCB 2 (3-

chlorobiphenyl-4-ol) and 4'-OH-PCB 2 (3'-chlorobiphenyl-4-ol), by CYP1A2 or CYP2D6. Besides, CYP2C19 and CYP3A4 may make minor contributions to the oxidation of PCB 2 in the *para* position. Similarly, ADMET Predictor predicted the oxidation of PCB 3 and PCB 11 by CYP2A2 and CYP2D6 in the *para* position.[1, 2] Experimental results with purified P450 enzymes also demonstrate that rat CYP1A2 metabolizes PCB 11 in the *para*-position.[6] In contrast, *ortho*-substituted PCBs are typically metabolized in *meta* position by rat CYP2B1[7, 8] and human CYP2B6 enzymes,[5] or in *para* position by CYP2A6.[5, 9, 10] CYP1A2 and CYP2D6 did not metabolize the *ortho*-chlorinated PCB congeners in these earlier metabolism studies. However, metabolism studies are needed to confirm that these P450 isoforms are involved in the metabolism of PCB 2 and other LC-PCBs.

Prediction of the biotransformation of PCB 2 with MetaDrug. MetaDrug predicted the formation of mono- and di-hydroxylated, sulfated, and glucuronidated PCB 2 metabolites. In addition, MetaDrug suggested the formation of PCB 2 arene oxide, dihydrodiols, dechlorinated glutathione adducts, and mono-methoxylated PCB 2 metabolites. MetaDrug predicted similar metabolites for the metabolism of PCB 3 and PCB 11.[1, 2] These predictions are consistent with the metabolites of other PCB congeners detected *in vitro*[5, 11, 12] or *in vivo*.[13-15] Dechlorinated glutathione and cysteine adducts (i.e., glutathione and cysteine adducts of BP) and mono-substituted methoxylated PCB 2 metabolites are an exception. These metabolites have not been detected experimentally in human-relevant models; however, dechlorinated OH-PCB metabolites have been reported in several animal models.[16-19]

Prediction of the biotransformation of PCB 2 metabolites. Human metabolism of selected PCB 2 metabolites, including mono-, di- or tri-hydroxylated, mono-sulfated, mono-glucuronidated, and methoxylated mono- or dihydroxylated metabolites, were also predicted with ADMET

Predictor and MetaDrug. The goal was to expand the candidate screening list for the Nt-HRMS analysis. ADMET Predictor suggested that all PCB 2 metabolites undergo biotransformation to metabolites with an additional hydroxyl group, likely involving oxidation by CYP1A2 or CYP2C9. ADMET Predictor also suggested the CYP-mediated formation of demethylated products (i.e., di- or tri-hydroxylated PCB 2 metabolites) from methoxylated and mono- or di-hydroxylated PCB 2 metabolites. Moreover, PCB 2 catechols were predicted to be oxidized to quinone metabolites. ADMET Predictor also suggested the formation of PCB 2 glucuronide metabolites by uridine 5'-diphospho-glucuronosyltransferases (UGTs).

MetaDrug suggested the formation of oxidized metabolites with additional hydroxyl groups and the corresponding methyl ester, sulfate, and glucuronide metabolites from the PCB 2 metabolites included in the predictions. In addition, MetaDrug predicted arene oxide metabolites and other metabolites formed via arene oxide intermediates from mono-, di- or tri-hydroxylated PCB 2 metabolites (e.g., dihydrodiol metabolites, dechlorinated cysteine, and glutathione adducts with or without additional hydroxylation). Consistent with the ADMET Predictor results, MetaDrug proposed catechol quinone metabolites for di- or tri-hydroxylated catechol metabolites as parent compounds. These quinone metabolites include quinones and hydroxy-, cysteine- and glutathione-quinones for di-OH-PCB 2, and mono- or di-hydroxy-, hydroxy-cysteine-, hydroxy-glutathione-, methoxy-, sulfate- and glucuronide-quinones for tri-OH-PCB 2 metabolites. No significant difference in the metabolism was suggested by MetaDrug when using different isomers of mono- or di-hydroxylated PCB 2 metabolites as parent compounds.

According to MetaDrug, PCB 2 sulfates and glucuronides are oxidized to metabolites with additional hydroxyl or epoxide groups and the corresponding downstream metabolites (e.g., methoxylated metabolites and sulfate and glucuronide conjugates derived from hydroxylated

metabolites, or dihydrodiol and dechlorinated cysteine and glutathione adducts derived from arene oxide metabolites). Methoxylated mono- or di-hydroxylated PCB 2 metabolites (i.e., MeO-OH-PCB 2 or MeO-di-OH-PCB 2) underwent demethylation, followed by the formation of sulfate metabolites of the corresponding di- or tri-hydroxylated PCB 2 metabolites, as described above. Also, methylated, sulfated, or glucuronidated metabolites were predicted by MetaDrug for these methoxylated and hydroxylated PCB 2 metabolites.

Metabolite identification and quality assurance/quality control. Extracted ion chromatograms are based on the calculated exact masses with a mass window of 10 ppm. PCB 2 metabolites are reported if they were detected in all replicate samples with an abundance > 10-times the background. The following criteria were used to identify PCB 2 metabolites: All differences between measured and calculated accurate mass were < 5 ppm. The isotopic mass pattern of the molecular ions was consistent with the pattern predicted for monochlorinated PCB 2 metabolites within a 20% error. The signal-to-noise ratio in the extracted ion chromatograms for all metabolites listed in Table 1 ranged from 214 to infinite, as implemented by the Xcalibur software. Whenever possible, MS/MS spectra were acquired for at least one isomer of each PCB 2 metabolite class to confirm key structural elements. 3-F-4'-OH-PCB 3 and 3-F-4'-PCB 3 sulfate were used to monitor the extraction and instrumental analysis performance. Control samples analyzed in parallel included blank incubations exposed to 0.1 % DMSO only and cell culture medium without cells and PCB 2. No metabolites were detected in any control sample.

Table S1. Summary of PCB 2 metabolites identified in cell culture medium from HepG2 cells exposed to 10 µM or 3.6 nM PCB 2 for 24 h. As part of an initial screening, extracts of the media from PCB 2-exposed and control incubations (DMSO only) were analyzed by LC-QTof MS.[a]

Class No	Metabolites	Retention time[b], min	Formula	Normalized intensity[c]		Accurate mass difference[e], ppm		Confidence level[f]
				High	Low[d]	High	Low[d]	
1.1	OH-PCB 2	8.46	$C_{12}H_8ClO^-$	124±2	17±3	10.3	13.8	3
1.2	PCB 2 sulfate	6.16	$C_{12}H_8ClSO_4^-$	5366±263	802±62	4.2	6.0	2
1.3	PCB 2 glucuronide	4.81	$C_{18}H_{16}ClO_7^-$	137±17	ND	0.4	ND	3
2.1	OH-PCB 2 sulfate	4.73	$C_{12}H_8ClSO_5^-$	137±8	ND	1.7	ND	2
3.1	MeO-OH-PCB 2	8.81	$C_{13}H_{10}ClO_2^-$	32±6	ND	10.4	ND	3
3.2	MeO-PCB 2 sulfate	5.77	$C_{13}H_{10}ClSO_5^-$	69±6	ND	3.6	ND	2
		6.06		63±7	ND	3.9	ND	3
		6.20		100±7	ND	2.6	ND	2
		6.38		231±3	42±2	2.2	3.3	2
3.3	MeO-PCB 2 glucuronide	4.94	$C_{19}H_{18}ClO_8^-$	24±3	ND	5.5	ND	3
4.2	MeO-BP sulfate	5.45	$C_{13}H_{11}SO_5^-$	38±7	ND	6.1	ND	3
		5.55		84±11	22±5	9.2	7.8	2

[a] Metabolites were extracted from the cell culture medium by QuEChERS extraction. Extracts were analyzed by LC-QTof MS. PCB 2 metabolites were also detected by LC-Orbitrap MS analyses of the same extracts (Table 1).
[b] Retention times were obtained on an LC-QTof MS with an Acquity UPLC BEH C18 column.
[c] For the calculation of normalized intensities, see the Experimental Section.
[d] ND, not detected.
[e] The accurate mass differences in parts per million (ppm) were calculated as the absolute value of (measured mass-calculated mass)/calculated mass × 10^6.
[f] Confidence levels for identifying PCB metabolites were assigned using the Schymanski framework.[20] Level 1: metabolites were not only identified based on accurate mass, isotope pattern, MS, and MS/MS, but also with authentic standards. Level 2: metabolites were identified based on accurate mass, isotope pattern, MS, and MS/MS. Level 3: metabolites were identified based on accurate mass, isotope pattern, and MS, but not MS/MS.

Table S2. Metabolism of PCB 2 and its metabolites by human cytochrome P450 enzymes and UDP-glucuronosyltransferases (UGTs) predicted by ADMET Predictor.

Starting compounds	P450 isoforms	Hydroxylated metabolites	Quinone metabolites	UGT isoforms	Glucuronide metabolites
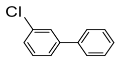	1A2 (17.2)<br>2A6<br>2B6<br>2C19 (<0.1)<br>2C8<br>2D6 (0.7)<br>2E1<br>3A4 (<0.1)	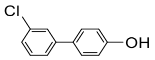			
	1A2 (13.3)<br>2A6<br>2B6<br>2C19 (<0.1)<br>2C8<br>2D6 (0.9)<br>2E1<br>3A4 (<0.1)	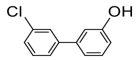			
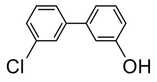	1A2 (25.2)<br>2C19 (0.3)<br>2C9 (22.7)<br>2D6 (0.1)	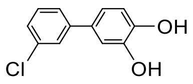	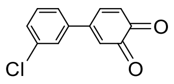	1A3<br>1A6<br>1A8<br>1A9<br>1A10<br>2B7<br>2B15	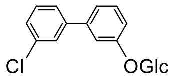
	1A2 (34.6)<br>2C19 (0.3)<br>2C9 (37.9)<br>2D6 (0.5)	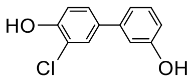			
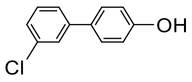	1A2 (17.7)	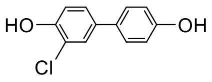		1A1<br>1A3<br>1A6<br>1A8<br>1A9<br>1A10<br>2B7<br>2B15	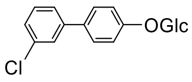
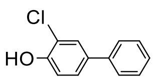	1A2 (28.9)<br>2C19 (0.3)<br>2C9 (31.9)<br>2D6 (0.7)	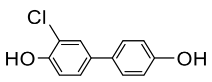		1A1<br>1A3<br>1A6<br>1A8<br>1A9<br>1A10<br>2B7<br>2B15	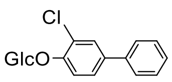

Glc: Glucuronide; deGlc: Dehydrogenated glucuronide. See the experimental section above for details about the metabolism predictions using ADMET Predictor.

Table S2 (Continued). Metabolism of PCB 2 and its metabolites by human cytochrome P450 enzymes and UDP-glucuronosyltransferases (UGTs) predicted by ADMET Predictor.

Starting compounds	P450 isoforms	Hydroxylated metabolites	Quinone metabolites	UGT isoforms	Glucuronide metabolites
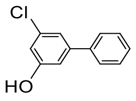	1A2 (25.1)<br>2C19 (0.3)<br>2D6 (<0.1)	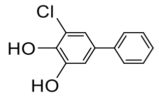	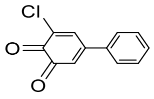	1A3<br>1A6<br>1A8<br>1A9<br>1A10<br>2B7<br>2B15	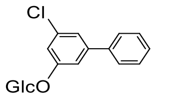
	1A2 (20.1)<br>2C19 (0.4)<br>2C9 (31.5)<br>2D6 (0.5)	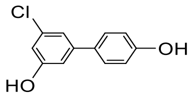			
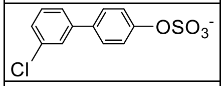	2C19 (<0.1)<br>2C9 (79.4)	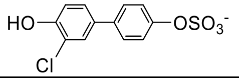			
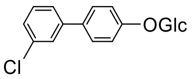	2C9 (17.1)	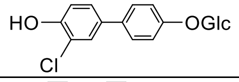		1A3<br>or<br>2B7	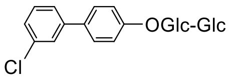
	2C9	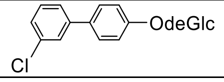			
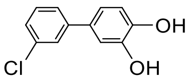	1A2 (4.9)<br>2C19 (1.6)<br>2C9 (79.5)<br>2D6 (0.8)	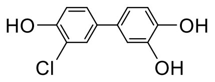	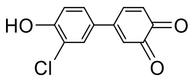	1A1<br>1A3<br>1A6<br>1A8<br>1A9<br>1A10<br>2B7<br>2B15	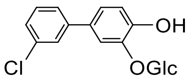
					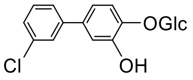
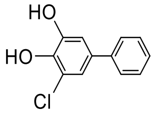	1A2 (2.9)<br>2C19 (2.0)<br>2C9 (55.3)	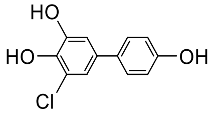		1A1<br>1A3<br>1A6<br>1A8<br>1A9<br>1A10<br>2B7<br>2B15	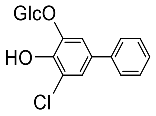
				1A3<br>1A6<br>1A8<br>1A9<br>1A10<br>2B7<br>2B15	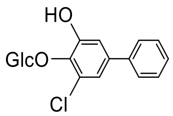

Glc: Glucuronide; deGlc: Dehydrogenated glucuronide. See the experimental section above for details about the metabolism predictions using ADMET Predictor.

Table S2 (Continued). Metabolism of PCB 2 and its metabolites by human cytochrome P450 enzymes and UDP-glucuronosyltransferases (UGTs) predicted by ADMET Predictor.

Starting compounds	P450 isoforms	Hydroxylated metabolites	Quinone metabolites	UGT isoforms	Glucuronide metabolites
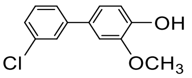	1A2 (115.3)<br>2C19 (1.4)<br>2C9 (46.7)<br>2D6 (0.1)	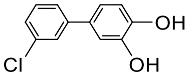	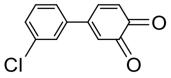	1A1<br>1A3<br>1A8<br>1A9<br>1A10<br>2B7<br>2B15	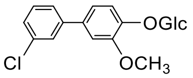
	1A2 (12.2)<br>2C19 (0.4)<br>2C9 (59.7)<br>2D6 (2.8)	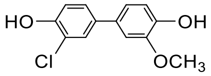			
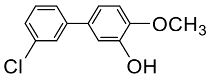	1A2 (121.8)<br>2C19 (2.0)<br>2C9 (67.9)<br>2D6 (0.1)	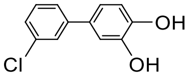	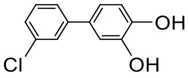	1A1<br>1A3<br>1A8<br>1A9<br>1A10<br>2B7<br>2B15	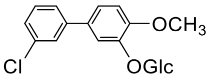
	1A2 (13.5)<br>2C19 (0.4)<br>2C9 (68.4)<br>2D6 (2.3)	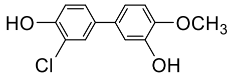			
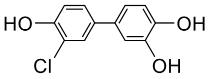	1A2 (0.4)	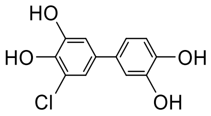	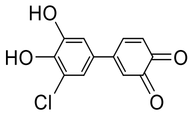	1A1<br>1A3<br>1A6<br>1A8<br>1A9<br>1A10<br>2B7	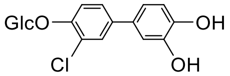<br>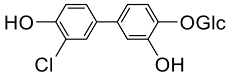<br>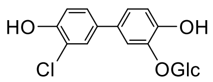
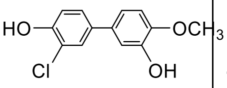	1A2 (63.8)<br>2C19 (5.2)<br>2C9 (105.5)<br>2D6 (0.1)	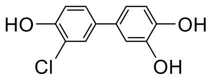	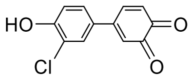	1A1<br>1A3<br>1A8<br>1A9<br>1A10<br>2B7<br>2B15	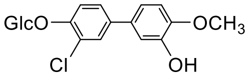<br>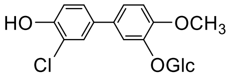
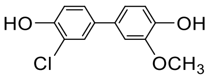	1A2 (63.8)<br>2C19 (5.2)<br>2C9 (105.5)<br>2D6 (0.1)	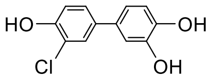	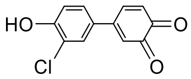	1A1<br>1A3<br>1A8<br>1A9<br>1A10<br>2B7<br>2B15	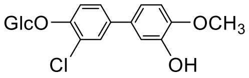<br>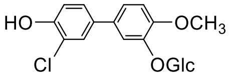

Glc: Glucuronide; deGlc: Dehydrogenated glucuronide. See the experimental section above for details about the metabolism predictions using ADMET Predictor.

Table S3. Metabolites formed from PCB 2 and its metabolites in humans, as predicted by MetaDrug.

Metabolites classes[a]	Parent compounds (abbreviations and the corresponding structures)													
	PCB 2	3'-OH-PCB 2	4'-OH-PCB 2	4-OH-PCB 2	5-OH-PCB 2	4'-PCB 2sulfate	4'-PCB 2 glucuronide	3',4'-di-OH-PCB 2	4,5-di-OH-PCB 2	3'-MeO-4'-OH-PCB 2	4'-MeO-3'-OH-PCB 2	3,4,4'-tri-OH-PCB 2	3'-MeO-4,4'-di-OH-PCB 2	4'-MeO-3',4-di-OH-PCB 2
	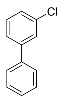	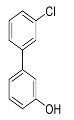	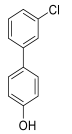	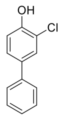	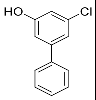	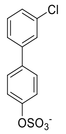	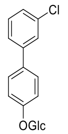	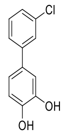	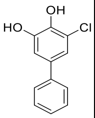	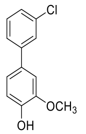	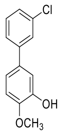	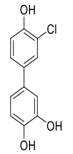	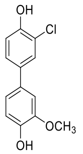	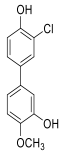
Mono-hydroxy	+													
Mono-Sulfate	+	+	+		+									
Mono-Glucuronide	+	+	+		+									
Methoxy	+	+	+											
Epoxide	+													
Cysteine[b]	+													
Glutathione[b]	+													
Dihydrodiol	+													
Hydroxy-dihydrodiol		+	+		+									
Dihydrodiol-Sulfate						+								
Dihydrodiol-glucuronide							+							
Di-hydroxy	+	+	+	+	+					+	+			
Monohydroxy-cysteine[b]	+	+	+		+									
Monohydroxy-glutathione[b]	+	+	+		+									
Quinone		+						+	+	+	+			

[a] Metabolite classes predicted according to MetaDrug from PCB 2 or its metabolites are indicated by “+”.
[b] Class of dechlorinated metabolites.
[c] Metabolite classes with dehydrogenated, methylated, sulfate, glucuronidated, cysteinylated, or glutathionylated glucuronide moieties with or without hydroxylation on the benzene rings.
Cells with a light blue background indicate the metabolite class was experimentally identified (Table 1 and Table S4).

Table S3 (Continued). Metabolites formed from PCB 2 and its metabolites in humans, as predicted by MetaDrug.

Metabolites classes[a]	Parent compounds (abbreviations and the corresponding structures)													
	PCB 2	3'-OH-PCB 2	4'-OH-PCB 2	4-OH-PCB 2	5-OH-PCB 2	4'-PCB 2sulfate	4'-PCB 2 glucuronide	3',4'-di-OH-PCB 2	4,5-di-OH-PCB 2	3'-MeO-4'-OH-PCB 2	4'-MeO-3'-OH-PCB 2	3,4,4'-tri-OH-PCB 2	3'-MeO-4,4'-di-OH-PCB 2	4'-MeO-3',4-di-OH-PCB 2
	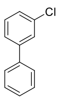	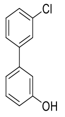	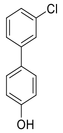	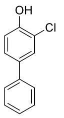	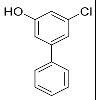	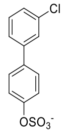	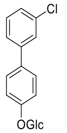	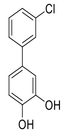	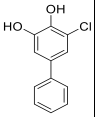	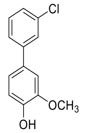	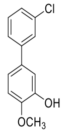	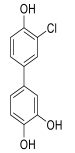	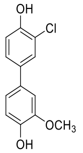	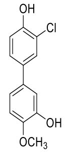
Monohydroxy-sulfate		+	+	+	+	+		+	+	+	+			
Monohydroxy-glucuronide		+	+	+	+		+	+	+	+	+			
Hydroxy-epoxide		+	+		+									
Tri-hydroxy		+	+	+	+			+	+	+	+		+	+
Dihydroxy-dihydrodiol								+	+					
Dihydroxy-cysteine		+	+	+	+			+	+	+	+			
Dihydroxy-glutathione		+	+	+	+			+	+	+	+			
Methoxy-hydroxy		+	+	+	+			+	+	+	+			
Dihydroxy-epoxide								+	+					
Dihydroxy-methoxy								+	+	+	+	+	+	+
Dihydroxy-sulfate						+		+	+			+	+	+
Dihydroxy-glucuronide							+	+	+			+	+	+
Tetra-hydroxy								+	+			+	+	+
Trihydroxy-methoxy										+	+		+	+
Hydroxy-epoxide		+	+		+									

[a] Metabolite classes predicted according to MetaDrug from PCB 2 or its metabolites are indicated by "+".
[b] Class of dechlorinated metabolites.
[c] Metabolite classes with dehydrogenated, methylated, sulfate, glucuronidated, cysteinylated, or glutathionylated glucuronide moieties with or without hydroxylation on the benzene rings.
Cells with a light blue background indicate the metabolite class was experimentally identified (Table 1 and Table S4).

Table S3 (Continued). Metabolites formed from PCB 2 and its metabolites in humans, as predicted by MetaDrug.

Metabolites classes[a]	Parent compounds (abbreviations and the corresponding structures)													
	PCB 2	3'-OH-PCB 2	4'-OH-PCB 2	4-OH-PCB 2	5-OH-PCB 2	4'-PCB 2sulfate	4'-PCB 2 glucuronide	3',4'-di-OH-PCB 2	4,5-di-OH-PCB 2	3'-MeO-4'-OH-PCB 2	4'-MeO-3'-OH-PCB 2	3,4,4'-tri-OH-PCB 2	3'-MeO-4,4'-di-OH-PCB 2	4'-MeO-3',4-di-OH-PCB 2
	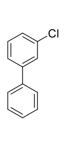	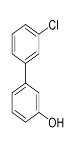	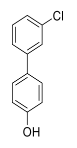	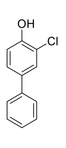	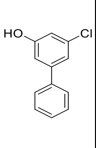	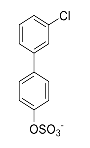	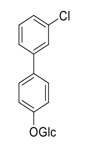	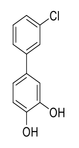	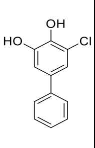	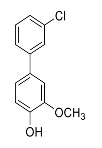	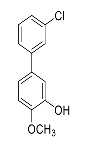	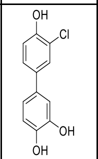	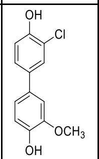	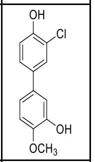
Trihydroxy-dihydrodiol												+		
Hydroxy-cysteine-sulfate[b]						+								
Hydroxy-glutathione-sulfate[b]						+								
Hydroxy-cysteine-glucuronide[b]							+							
Hydroxy-glutathione-glucuronide[b]							+							
Sulfate-glucuronide						+	+							
Methoxy-sulfate						+				+	+			
Methoxy-glucuronide							+			+	+			
Hydroxy-methoxy-sulfate										+	+		+	+
Hydroxy-methoxy-glucuronide										+	+		+	+
Di-sulfate						+								
Di-glucuronide							+							
Epoxide-sulfate						+								
Epoxide-glucuronide							+							
Dimethoxy										+	+			

[a] Metabolite classes predicted according to MetaDrug from PCB 2 or its metabolites are indicated by "+".
[b] Class of dechlorinated metabolites.
[c] Metabolite classes with dehydrogenated, methylated, sulfate, glucuronidated, cysteinylated, or glutathionylated glucuronide moieties with or without hydroxylation on the benzene rings.
Cells with a light blue background indicate the metabolite class was experimentally identified (Table 1 and Table S4).

Table S3 (Continued). Metabolites formed from PCB 2 and its metabolites in humans, as predicted by MetaDrug.

Metabolites classes[a]	Parent compounds (abbreviations and the corresponding structures)													
	PCB 2	3'-OH-PCB 2	4'-OH-PCB 2	4-OH-PCB 2	5-OH-PCB 2	4'-PCB 2sulfate	4'-PCB 2 glucuronide	3',4'-di-OH-PCB 2	4,5-di-OH-PCB 2	3'-MeO-4'-OH-PCB 2	4'-MeO-3'-OH-PCB 2	3,4,4'-tri-OH-PCB 2	3'-MeO-4,4'-di-OH-PCB 2	4'-MeO-3',4-di-OH-PCB 2
	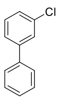	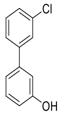	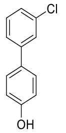	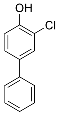	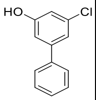	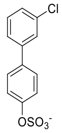	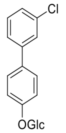	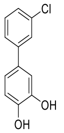	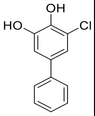	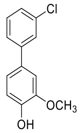	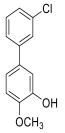	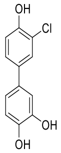	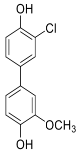	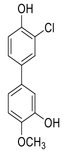
Hydroxy-dimethoxy										+	+		+	+
Methoxy-hydroxy-epoxide										+	+			
Methoxy-dihydroxy-epoxide													+	+
Methoxy-hydroxy-dihydrodiol										+	+			
Methoxy-dihydroxy-dihydrodiol													+	+
Methoxy-hydroxy-cysteine[b]										+	+			
Methoxy-hydroxy-glutathione[b]										+	+			
Methoxy-dihydroxy-cysteine[b]										+	+		+	+
Methoxy-dihydroxy-glutathione[b]										+	+		+	+
Cysteine-sulfate						+								
Glutathione-sulfate						+								
Cysteine-glucuronide							+							
Glutathione-glucuronide							+							
Dehydrogenated glucuronide[c]							+							
Methylated glucuronide[c]							+							

[a] Metabolite classes predicted according to MetaDrug from PCB 2 or its metabolites are indicated by "+".
[b] Class of dechlorinated metabolites.
[c] Metabolite classes with dehydrogenated, methylated, sulfate, glucuronidated, cysteinylated, or glutathionylated glucuronide moieties with or without hydroxylation on the benzene rings.
Cells with a light blue background indicate the metabolite class was experimentally identified (Table 1 and Table S4).

Table S3 (Continued). Metabolites formed from PCB 2 and its metabolites in humans, as predicted by MetaDrug.

Metabolites classes[a]	Parent compounds (abbreviations and the corresponding structures)													
	PCB 2	3'-OH-PCB 2	4'-OH-PCB 2	4-OH-PCB 2	5-OH-PCB 2	4'-PCB 2sulfate	4'-PCB 2 glucuronide	3',4'-di-OH-PCB 2	4,5-di-OH-PCB 2	3'-MeO-4'-OH-PCB 2	4'-MeO-3'-OH-PCB 2	3,4,4'-tri-OH-PCB 2	3'-MeO-4,4'-di-OH-PCB 2	4'-MeO-3',4-di-OH-PCB 2
	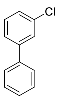	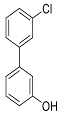	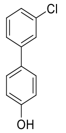	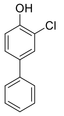	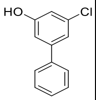	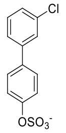	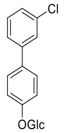	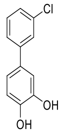	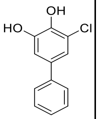	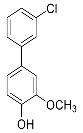	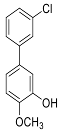	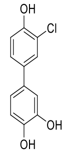	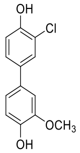	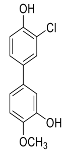
Sulfated glucuronide[c]							+							
Glucuronidated glucuronide[c]							+							
Cysteinylated glucuronide[c]							+							
Glutathionylated glucuronide[c]							+							
Hydroxy-quinone								+	+			+	+	+
Dihydroxy-quinone												+		
Cysteine-quinone								+	+					
Hydroxy-cysteine-quinone												+		
Glutathione-quinone								+	+					
Hydroxy-glutathione-quinone												+		
Methoxy-quinone												+		
Quinone glucuronide												+		
Quinone sulfate												+		

[a] Metabolite classes predicted according to MetaDrug from PCB 2 or its metabolites are indicated by "+".
[b] Class of dechlorinated metabolites.
[c] Metabolite classes with dehydrogenated, methylated, sulfate, glucuronidated, cysteinylated, or glutathionylated glucuronide moieties with or without hydroxylation on the benzene rings.
Cells with a light blue background indicate the metabolite class was experimentally identified (Table 1 and Table S4).

Table S4. The limits of detection (LODs) of mono- to tetra-chlorinated PCB sulfates analyzed by LC-QTof MS.[a]

PCB sulfates	Linear range, ng/mL	R^2	ΔI	S	LODs,[b] ng/mL
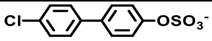	1 - 500	0.9998	0.44	0.26	5
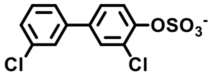	1 - 500	0.9998	0.26	0.14	6
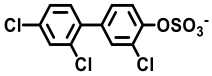	1 - 500	0.9994	0.36	0.12	9
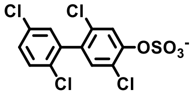	1 - 500	0.9999	0.24	0.22	3

[a] LC-QTof MS analyses were performed with five standard solutions of each mono- to tetra-chlorinated PCB sulfate at concentration levels ranging from 1 to 500 ng/mL.

[b] The LODs were calculated from derivations of intercepts (ΔI) of standard calibration curves and the slopes (S) of standard calibration curves with the equation: $LOD = 3 \times \Delta I/S$, as described.[21]

Table S5. Several likely PCB 2 metabolite classes (i.e., monochlorinated PCB metabolites) were detected by LC-Orbitrap MS analysis in HepG2 cell culture medium exposed to 10 µM PCB 11.[a]

Class No	Metabolites	Retention time[b], min	Formula $[M-H]^-$	$[M-H]^-$ Calculated (Da)	Measured (Da)	Differences (ppm)	MS/MS (Da)	Confidence level[c]
1	PCB 2 sulfate	4.15	$C_{12}H_8ClSO_4^-$	282.98320	282.98392	2.62	203.03	2
		4.22			282.98383	2.30	203.03	2
2	di-OH-PCB 2	4.53	$C_{12}H_8ClO_2^-$	219.02129	219.02220	0.09	191.03, 175.03	2
3	OH-PCB 2 sulfate	4.23	$C_{12}H_8ClSO_5^-$	298.97812	298.97879	2.31	219.02	2
4	OH-PCB 2 cysteine	4.02	$C_{15}H_{13}ClNSO_3^-$	322.03048	322.03122	2.30		3
5	MeO-OH-PCB 2	5.59	$C_{13}H_{10}ClO_2^-$	233.03694	233.03775	3.52	218.01	2
6	MeO-PCB 2 sulfate	4.19	$C_{13}H_{10}ClSO_5^-$	312.99377	312.99469	3.00	218.01, 79.96	2
		4.24			312.99457	2.62	233.04, 218.01	2
7	MeO-di-OH-PCB 2	4.69	$C_{13}H_{10}ClO_3^-$	249.03186	249.03262	3.09	234.01,198.03, 170.04,145.03	2
8	MeO-OH-PCB 2 sulfate	3.97	$C_{13}H_{10}ClSO_6^-$	328.98869	328.98953	2.64	249.03, 234.01	2
		4.01			328.98953	2.64	249.03, 234.01,	2

[a] Likely PCB 2 metabolites were detected in our earlier study investigating the metabolism of PCB 11.[2] Briefly, HepG2 cells were exposed for 24 h to PCB 11 (10 µM) with PCB 2 as an impurity (3.63 nM) as described,[22, 23] metabolites were extracted from the cell culture medium by QuEChERS extraction, and extracts were analyzed by LC-Orbitrap MS. The corresponding MS and MS/MS spectra are provided in Figs. S16-S20. These metabolites were attributed to the PCB 2 impurity and were not analyzed further in our earlier study.

[b] Injections for both LC-MS and MS/MS analysis were performed on an LC-Orbitrap MS with an Acquity UPLC BEH C18 column. The retention times were corrected for batch shifts using the internal standard (i.e., 3-F,4-PCB 3 sulfate).

[c] Confidence levels for identifying PCB metabolites were assigned using the Schymanski framework [20]. Level 1: metabolites were not only identified based on accurate mass, isotope pattern, MS, and MS/MS but also with authentic standards. Level 2: metabolites were identified based on accurate mass, isotope pattern, MS, and MS/MS. Level 3: metabolites were identified based on accurate mass, isotope pattern, and MS, but not MS/MS.

Table S6. Summary of BP or PCB 2 metabolites identified in HLM incubation with 10 µM PCB 2, 4-OH-PCB 2 or 3-OH-PCB 3 for 15 min.[a]

Parent compounds	Metabolites	Retention time[b], min	Formula $[M-H]^-$	$[M-H]^-$ Calculated (Da)	Measured (Da)	Differences (ppm)	MS/MS (Da)	Confidence level[c]
PCB 2	OH-PCB 2	6.73	$C_{12}H_8ClO^-$	203.02637	203.02635	0.10		3
	di-OH-PCB 2	6.17	$C_{12}H_8ClO_2^-$	219.02129	219.02151	1.00		3
4-OH-PCB 2	di-OH-BP	5.62	$C_{12}H_9O_2^-$	185.06027	185.05988	2.11	167.05,156.95	2
	di-OH-PCB 2	6.17	$C_{12}H_8ClO_2^-$	219.02129	219.02148	0.88	200.02, 155.05	2
3-OH-PCB 3	di-OH-BP	5.62	$C_{12}H_9O_2^-$	185.06027	185.05994	1.78		3
	di-OH-PCB 3	6.34	$C_{12}H_8ClO_2^-$	219.02129	219.02138	0.41		3

[a] BP or PCB 2 metabolites were extracted from the media of the HLM incubations by QuEChERS extraction. Extracts were analyzed by LC-Orbitrap MS with an Acquity UPLC BEH C18 column. See the text above for more details regarding metabolite extraction and instrumental analyses.

[b] The retention times were corrected for batch shifts using the internal standard (i.e., 3-F,4-PCB 3 sulfate).

[c] Confidence levels for identifying PCB metabolites were assigned using the Schymanski framework.[20] Level 1: metabolites were not only identified based on accurate mass, isotope pattern, MS, and MS/MS but also with authentic standards. Level 2: metabolites were identified based on accurate mass, isotope pattern, MS, and MS/MS. Level 3: metabolites were identified based on accurate mass, isotope pattern, and MS, but not MS/MS.

Table S7. The *m/z*, retention times, p-values, and confidence levels of the metabolites in the bile acid biosynthesis pathway identified through the metabolomic analysis for HepG2 cells exposed to high and low concentrations of PCB 2 or vehicle for 24 h (see Fig. 6).

Metabolites[a]	Molecular ion	*m*/*z*	Retention time [s]	p-value[b]	Confidence level[c]
Trihydroxycholestanal	$[M-H]^-$	433.3329	350.3	0.0373	3
Dihydroxycholestanate	$[M-H]^-$	433.3328	570.5	0.6408	3
Cholate	$[M-H]^-$	407.2809	237.1	0.0232	2
Glycocholate	$[M-H]^-$	464.3035	243.6	0.3823	2
Taurocholate	$[M-H]^-$	514.2846	237.7	0.0262	2
Chenodeoxycholate	$[M-H]^-$	391.2861	270.0	0.7712	3
Glycochenodeoxycholate	$[M-H]^-$	448.3077	247.9	0.0165	3
Taurochenodeoxycholate	$[M-H]^-$	498.2920	252.2	0.0601	2

[a] The metabolites were annotated with xMSannotator based on the Human Metabolome DataBase (HMDB)[24] and the Kyoto Encyclopedia of Genes and Genomes (KEGG).[25]

[b] The *p* values were obtained from linear regress on the normalized raw intensities of primary molecular ion $[M-H]^-$ with PCB concentrations in the cell culture experiments.

[c] The confidence levels were assigned by the *xMSannotator* software mainly based on the number of adduct ions and isotopic ions detected for the molecular ions. Level 3 and level 2 represent high and medium confidence, respectively. Only those features annotated with high or medium confidence levels are listed.[26]

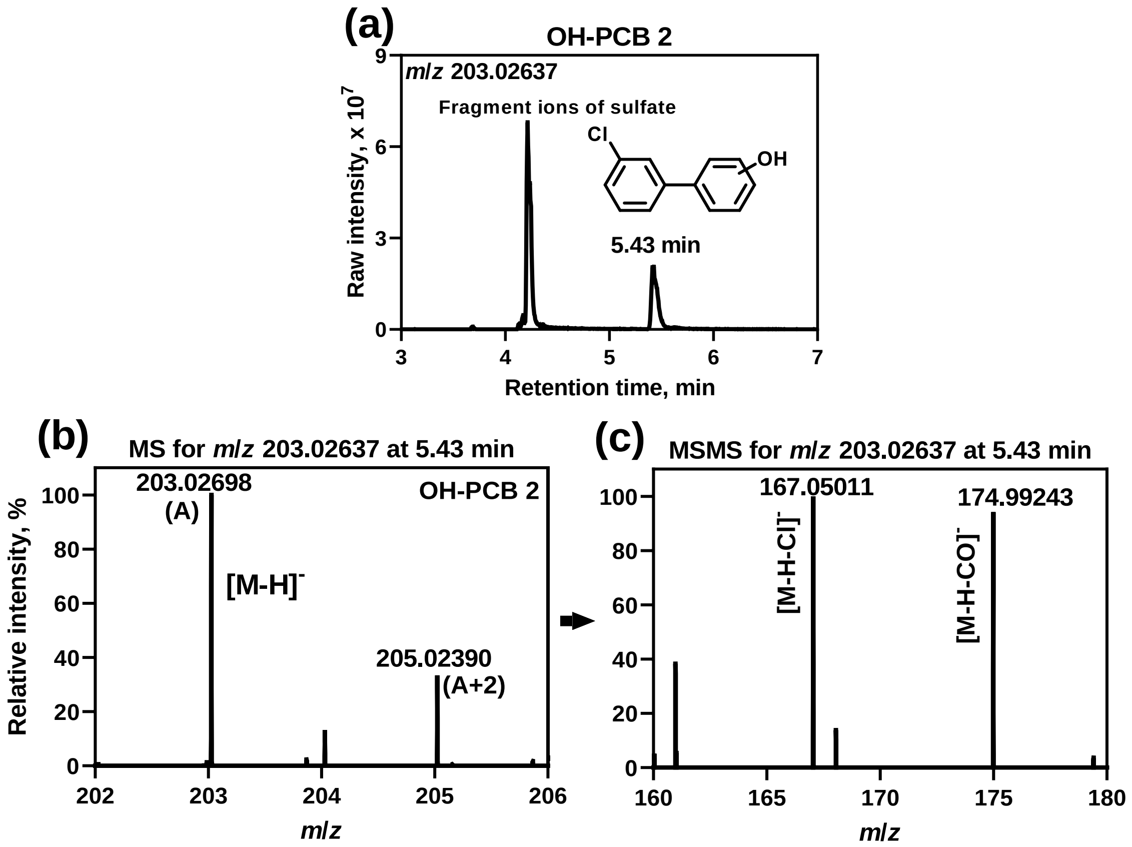

Fig. S1. OH-PCB 2 was present in the cell culture medium from HepG2 cells exposed to 10 µM PCB 2. (a) The extracted ion chromatogram (EIC) at *m/z* 203.02637 with a mass window of 10 ppm revealed the presence of OH-PCB 2 eluting at 5.43 min. The formation of OH-PCB 2 metabolite was further confirmed by the accurate mass of the molecular ion and its isotope pattern [panels (b)]. Furthermore, the MS/MS spectra of this PCB 2 metabolite showed fragment ions consistent with an OH-PCB 2 metabolite [panels (c)]. LC-MS and MS/MS analyses were performed on an LC-Orbitrap MS with an Acquity UPLC BEH C18 column. The collision energy of the MS/MS spectrum was 30 eV. For information about the formation of OH-PCB 2 in the cell culture medium from HepG2 cells exposed to 3.6 nM PCB 2, see Table 1.

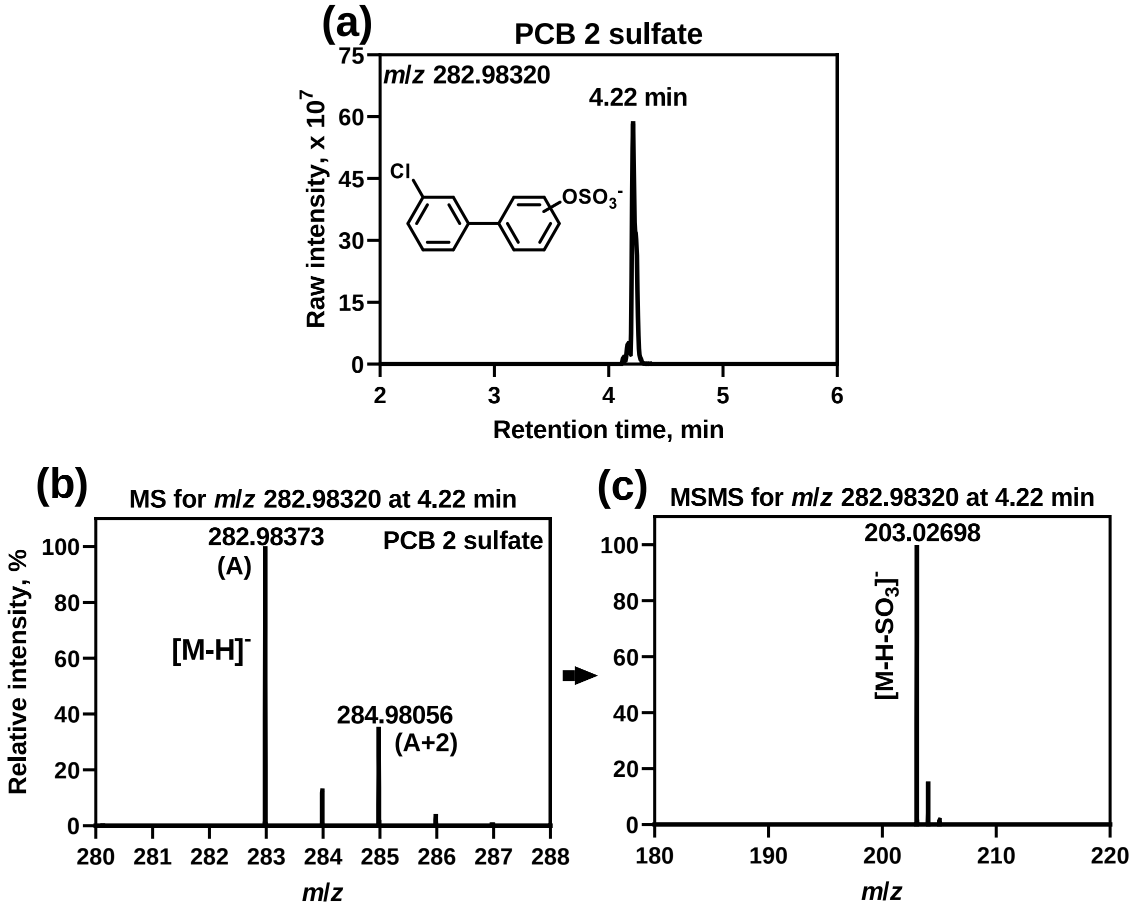

Fig. S2. PCB 2 sulfate was present in the cell culture medium from HepG2 cells exposed to 10 µM PCB 2. (a) The extracted ion chromatogram (EIC) at *m/z* 282.98320 with a mass window of 10 ppm revealed the presence of PCB 2 sulfate eluting at 4.22 min. The formation of PCB 2 sulfate metabolite was further confirmed by the accurate mass of the molecular ion and its isotope pattern [panels (b)]. Furthermore, the MS/MS spectra of this PCB 2 metabolite showed fragment ions consistent with PCB 2 sulfate [panels (c)]. LC-MS and MS/MS analyses were performed on an LC-Orbitrap MS with an Acquity UPLC BEH C18 column. The collision energy of the MS/MS spectrum was 30 eV. For information about the formation of PCB 2 sulfate in the cell culture medium from HepG2 cells exposed to 3.6 nM PCB 2, see Table 1.

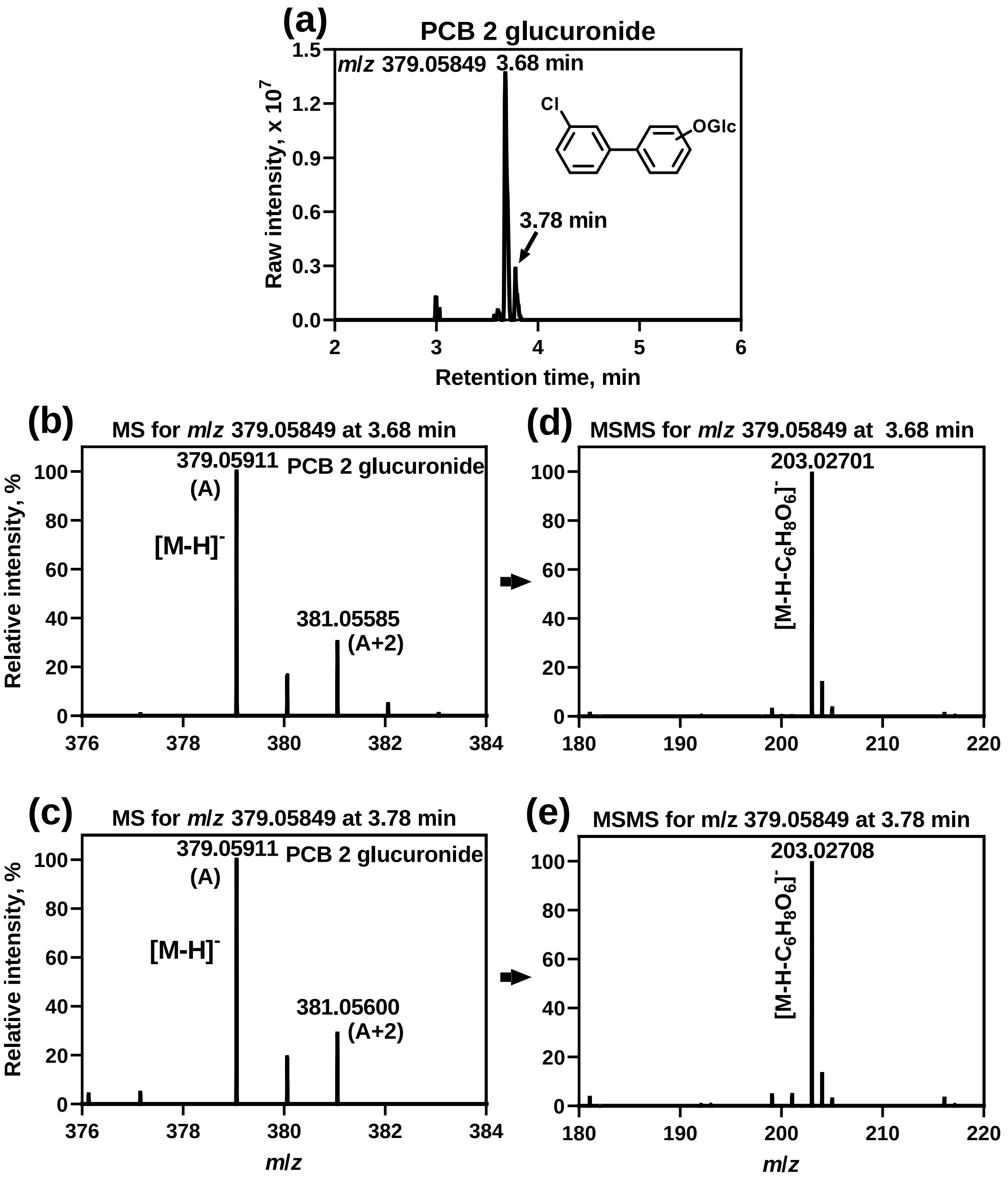

Fig. S3. Two PCB 2 glucuronide isomers were present in the cell culture medium from HepG2 cells exposed to 10 µM PCB 2. (a) The extracted ion chromatogram (EIC) at *m/z* 379.05849 with a mass window of 10 ppm revealed the presence of two PCB 2 glucuronide isomers eluting at 3.68 and 3.78 min. The formation of PCB 2 glucuronide metabolites was further confirmed by the accurate mass of the molecular ion and its isotope pattern [panels (b) and (c)]. Furthermore, the MS/MS spectra of both PCB 2 metabolites showed fragment ions consistent with PCB 2 glucuronides [panels (d) and (e)]. LC-MS and MS/MS analysis were performed on an LC-Orbitrap MS with an Acquity UPLC BEH C18 column. The collision energy of the MS/MS spectrum was 30 eV. For information about the formation of PCB 2 glucuronide in the cell culture medium from HepG2 cells exposed to 3.6 nM PCB 2, see Table 1.

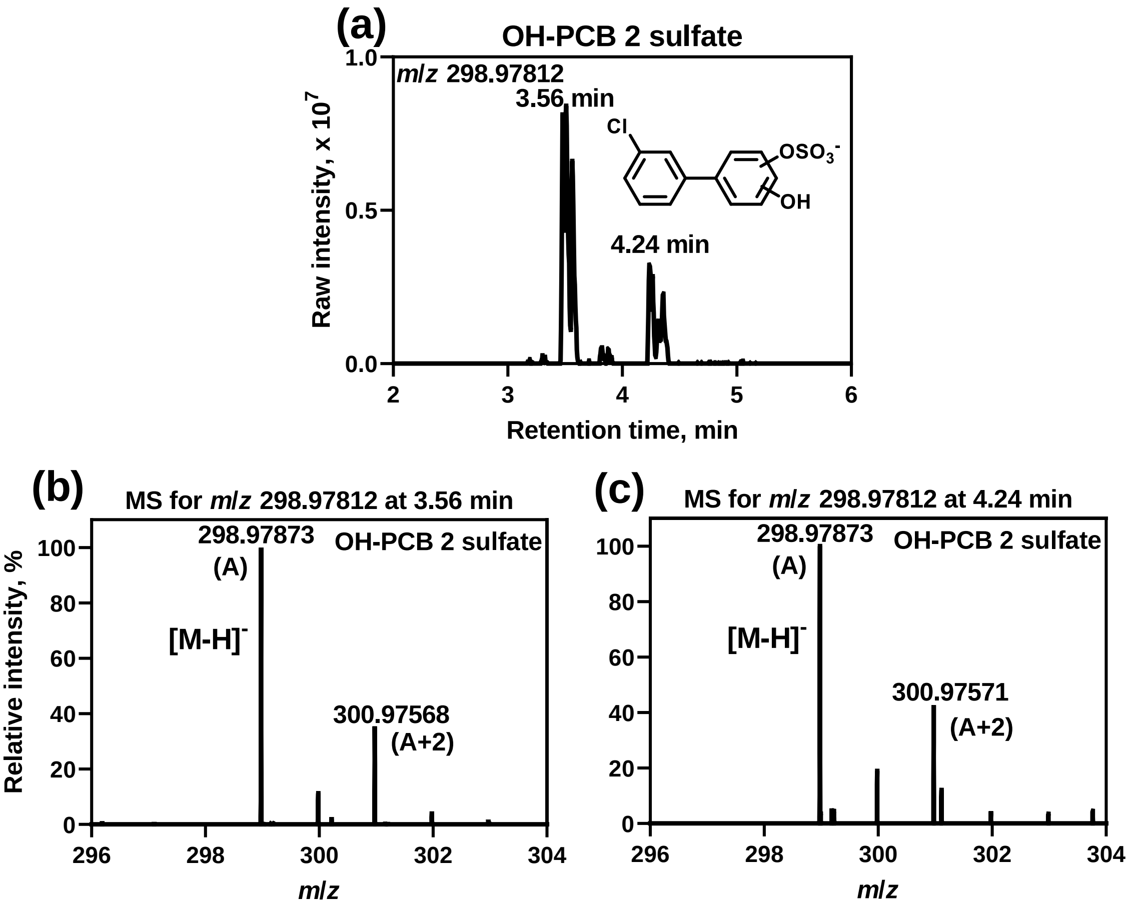

Fig. S4. Two OH-PCB 2 sulfates were present in the cell culture medium from HepG2 cells exposed to 10 µM PCB 2. (a) The extracted ion chromatogram (EIC) at *m/z* 298.97812 with a mass window of 10 ppm revealed the presence of OH-PCB 2 sulfate eluting at 3.56 and 4.23 min. The formation of OH-PCB 2 sulfate metabolite was further confirmed by the accurate mass of the molecular ion and its isotope pattern [panel (b) and (c) for isomers eluting at 3.56 and 4.24 min, respectively]. LC-MS and MS/MS analysis were performed on an LC-Orbitrap MS with an Acquity UPLC BEH C18 column. The collision energy of the MS/MS spectrum was 30 eV. For information about the formation of OH-PCB 2 sulfate in the cell culture medium from HepG2 cells exposed to 3.6 nM PCB 2, see Table 1.

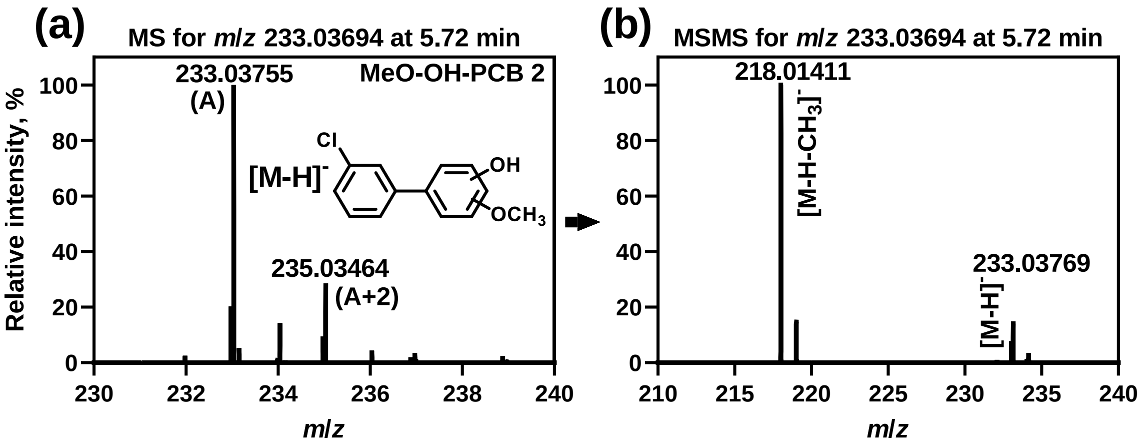

Fig. S5. One MeO-OH-PCB 2 metabolite (eluting at 5.72 min) was present in the cell culture medium from HepG2 cells exposed to 10 µM PCB 2. The identification of this metabolite was supported by (a) the MS spectrum showing the accurate mass of the molecular ion and its isotope pattern and (b) the MS/MS spectra showing fragment ions consistent with MeO-OH-PCB 2. LC-MS and MS/MS analysis were performed on an LC-Orbitrap MS with an Acquity UPLC BEH C18 column. The collision energy of the MS/MS spectrum was 30 eV. For the extracted ion chromatogram for MeO-OH-PCB 2 at *m/z* 233.03694, see Fig. 1. For information on the formation of MeO-OH-PCB 2 in the cell culture medium from HepG2 cells exposed to 3.6 nM PCB 2, see Table 1.

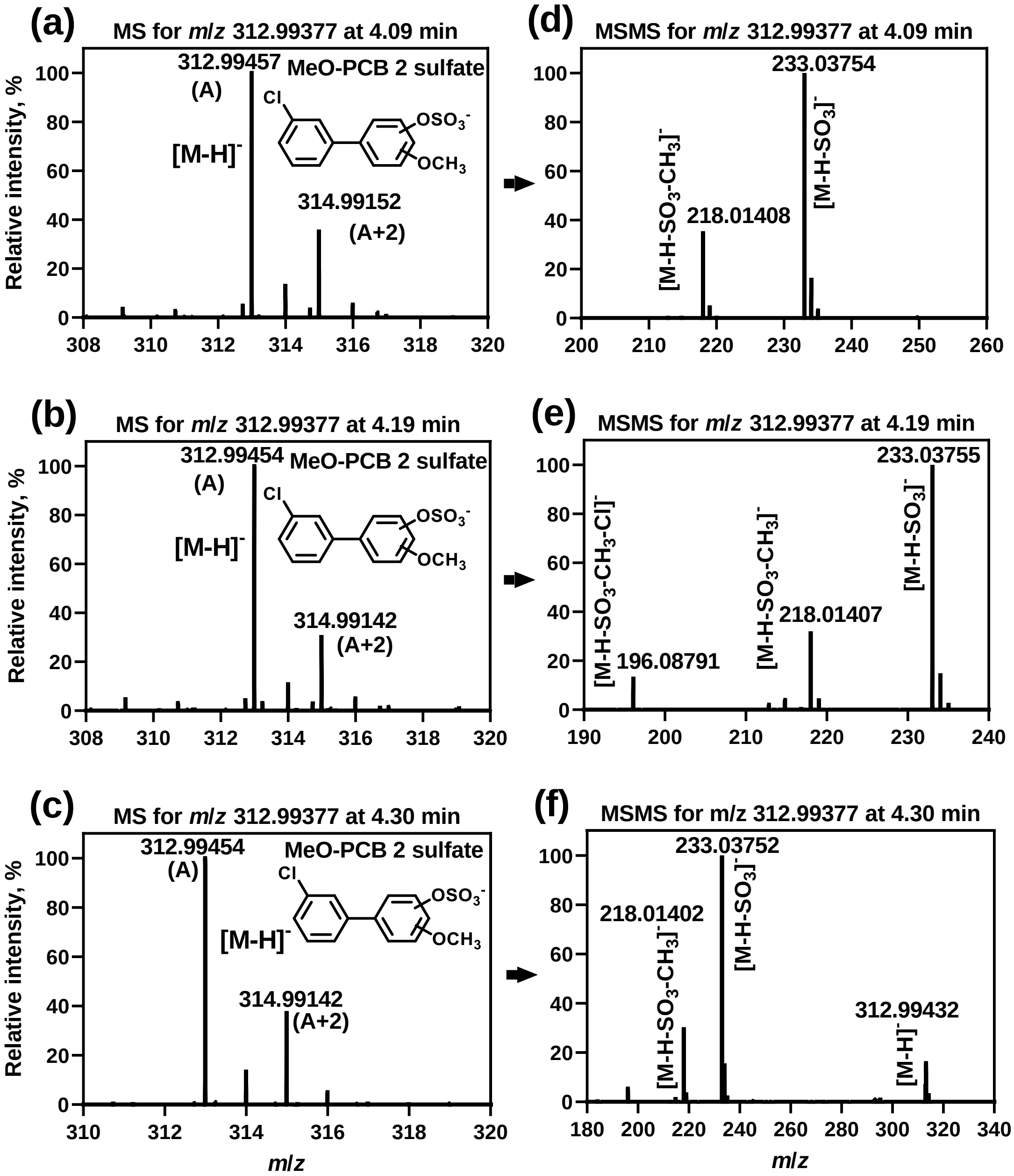

Fig. S6. Three MeO-PCB 2 sulfate isomers (eluting at 4.09, 4.19, and 4.30 min) were present in the cell culture medium from HepG2 cells exposed to 10 µM PCB 2. The identification of both metabolites was supported by MS spectra showing the accurate mass of the molecular ion and their isotope patterns [panel (a), (b), and (c) for isomers eluting at 4.09, 4.19 and 4.30 min, respectively] and the MS/MS spectra showing fragment ions consistent with MeO-PCB 2 sulfate [panel (d), (e) and (f) for isomers eluting at 4.09, 4.19 and 4.30 min, respectively]. LC-MS and MS/MS analysis were performed on an LC-Orbitrap MS with an Acquity UPLC BEH C18 column. The collision energy of the MS/MS spectra was 30 eV. For the extracted ion chromatogram for MeO-PCB 2 sulfate at *m/z* 312.99377, see Fig. 1. For information about the formation of MeO-PCB 2 sulfate in the cell culture medium from HepG2 cells exposed to 3.6 nM PCB 2, see Table 1.

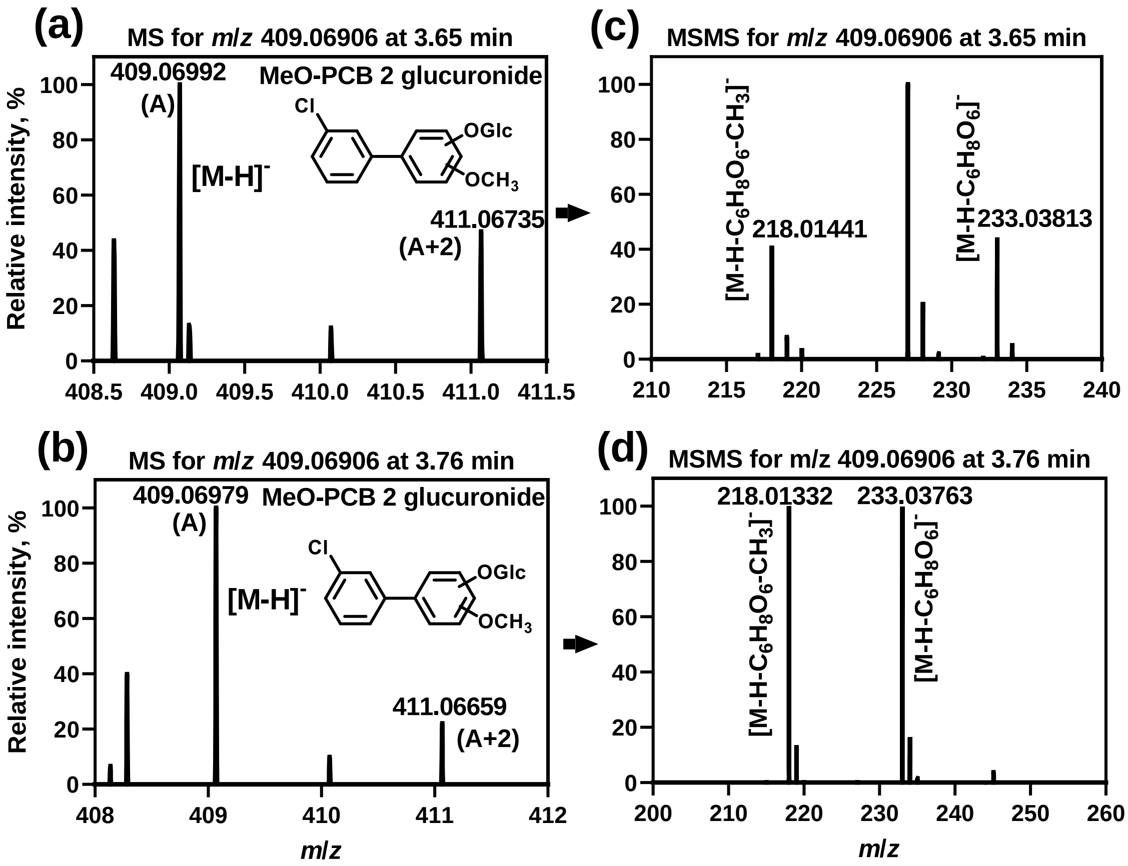

Fig. S7. Two MeO-PCB 2 glucuronide isomers (eluting at 3.65 and 3.76 min) were present in the cell culture medium from HepG2 cells exposed to 10 µM PCB 2. The identification of both metabolites was supported by MS spectra showing the accurate mass of the molecular ion and their isotope patterns [panel (a) and (b) for isomers eluting at 3.65 and 3.76 min, respectively] and the MS/MS spectra showing fragment ions consistent with MeO-PCB 2 glucuronide [panel (c) and (d) for isomers eluting at 3.65 and 3.76 min, respectively]. LC-MS and MS/MS analysis were performed on an LC-Orbitrap MS with an Acquity UPLC BEH C18 column. The collision energy of the MS/MS spectra was 30 eV. For the extracted ion chromatogram for MeO-PCB 2 glucuronide at *m/z* 409.06906, see Fig. 1. For information about the formation of MeO-PCB 2 glucuronide in the cell culture medium from HepG2 cells exposed to 3.6 nM PCB 2, see Table 1.

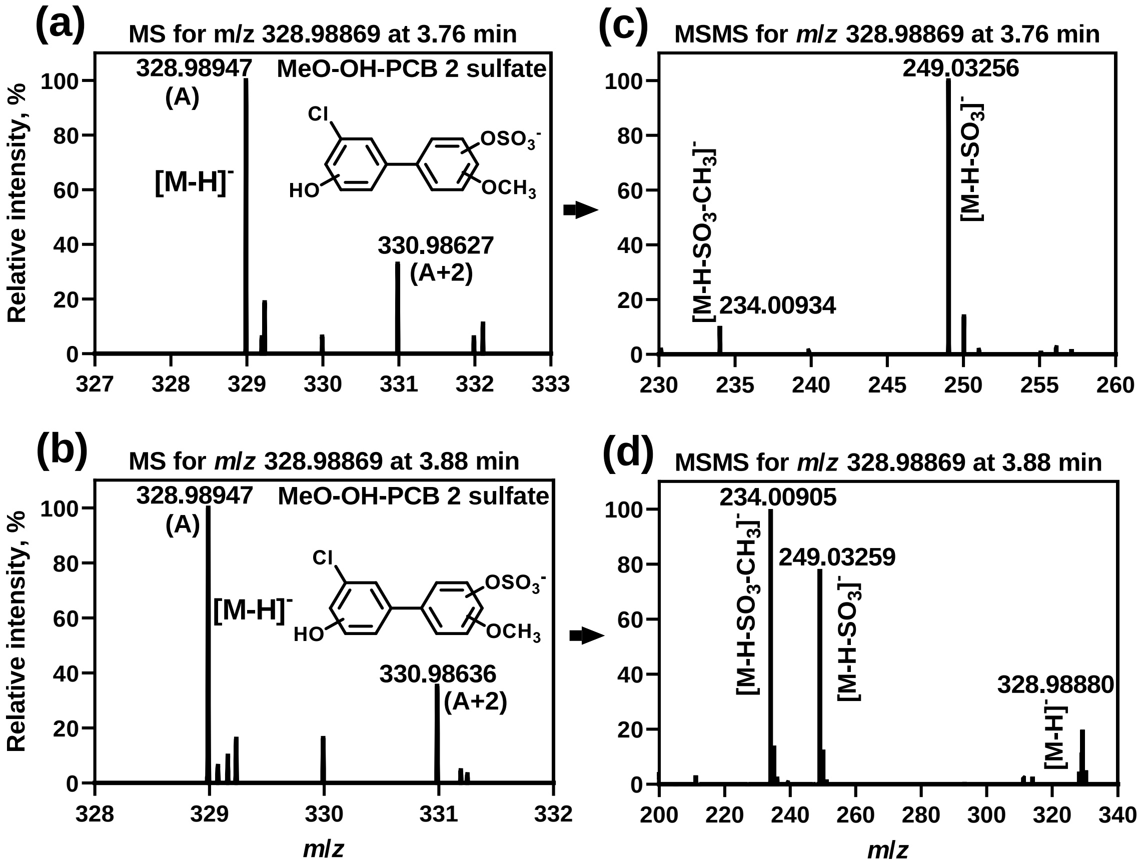

Fig. S8. Two MeO-OH-PCB 2 sulfate isomers (eluting at 3.76 and 3.88 min) were present in the cell culture medium from HepG2 cells exposed to 10 µM PCB 2. The identification of both metabolites was supported by MS spectra showing the accurate mass of the molecular ion and their isotope patterns [panel (a) and (b) for isomers eluting at 3.76 and 3.88 min, respectively] and the MS/MS spectra showing fragment ions consistent with MeO-OH-PCB 2 sulfate [panel (c) and (d) for isomers eluting at 3.76 and 3.88 min, respectively]. LC-MS and MS/MS analysis were performed on an LC-Orbitrap MS with an Acquity UPLC BEH C18 column. The collision energy of the MS/MS spectra was 30 eV. For the extracted ion chromatogram for MeO-OH-PCB 2 sulfate at *m/z* 328,98869, see Fig. 1. For information about the formation of MeO-OH-PCB 2 sulfate in the cell culture medium from HepG2 cells exposed to 3.6 nM PCB 2, see Table 1.

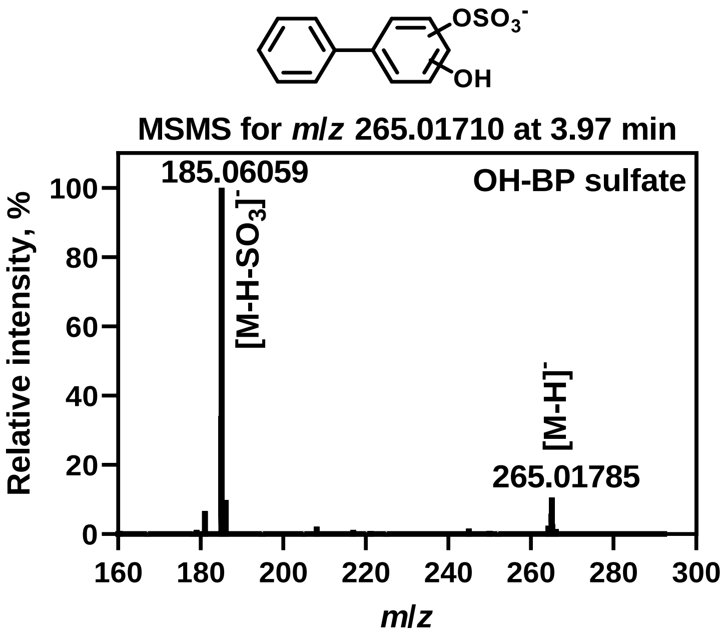

Fig. S9. One OH-BP sulfate metabolite (eluting at 3.97 min) was present in the cell culture medium from HepG2 cells exposed to 10 µM PCB 2. The identification of this metabolite was supported by the MS/MS spectrum showing fragment ions consistent with OH-BP sulfate. MS/MS analyses were performed on an LC-Orbitrap MS with an Acquity UPLC BEH C18 column. For the extracted ion chromatogram for OH-BP sulfate at *m/z* 265.01710, see Fig. 1. For information about the formation of OH-BP sulfate in the cell culture medium from HepG2 cells exposed to 3.6 nM PCB 2, see Table 1.

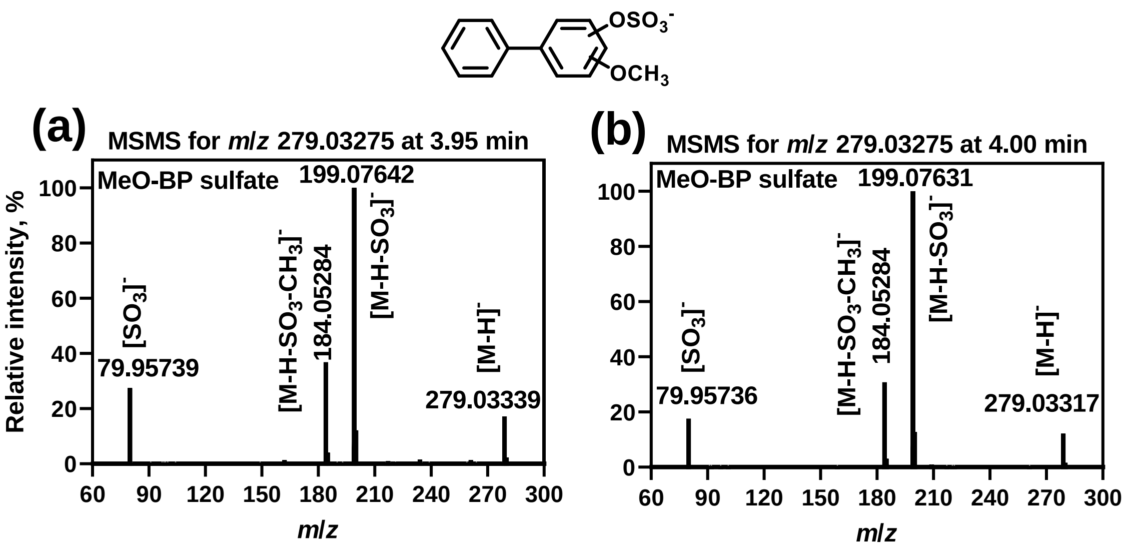

Fig. S10. Three MeO-BP sulfate isomers (eluting at 3.95 and 4.00 min) were present in the cell culture medium from HepG2 cells exposed to 10 µM PCB 2. The identification of this metabolite was supported by the MS/MS spectrum showing fragment ions consistent with MeO-BP sulfate [panel (a) and (b) for isomers eluting at 3.95 and 4.00 min, respectively]. MS/MS analyses were performed on an LC-Orbitrap MS with an Acquity UPLC BEH C18 column. For the extracted ion chromatogram for OH-BP sulfate at *m/z* 279.03275, see Fig. 1. For information about the formation of MeO-BP sulfate in the cell culture medium from HepG2 cells exposed to 3.6 nM PCB 2, see Table 1.

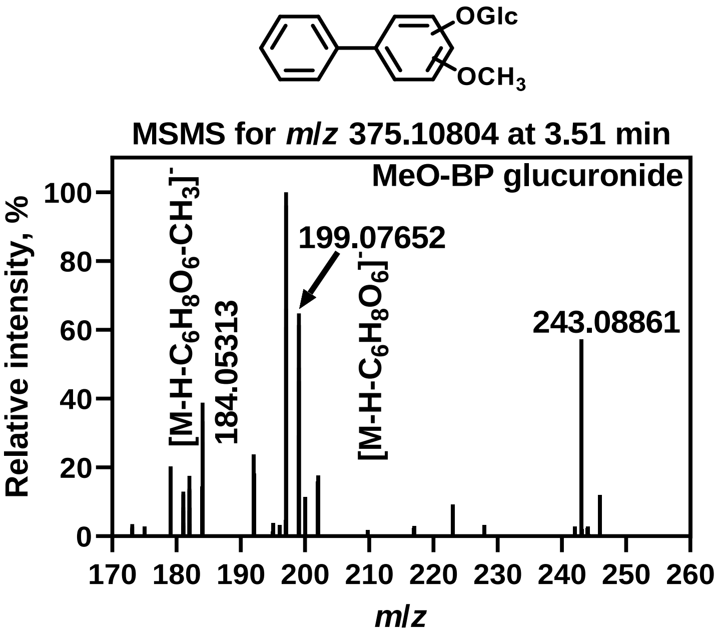

Fig. S11. One MeO-BP glucuronide metabolite (eluting at 3.51 min) was present in the cell culture medium from HepG2 cells exposed to 10 µM PCB 2. The identification of this metabolite was supported by the MS/MS spectrum showing fragment ions consistent with MeO-BP glucuronide. MS/MS analyses were performed on an LC-Orbitrap MS with an Acquity UPLC BEH C18 column. For the extracted ion chromatogram for MeO-BP glucuronide at *m/z* 375.10804, see Fig. 1. For information about the formation of MeO-BP glucuronide in the cell culture medium from HepG2 cells exposed to 3.6 nM PCB 2, see Table 1.

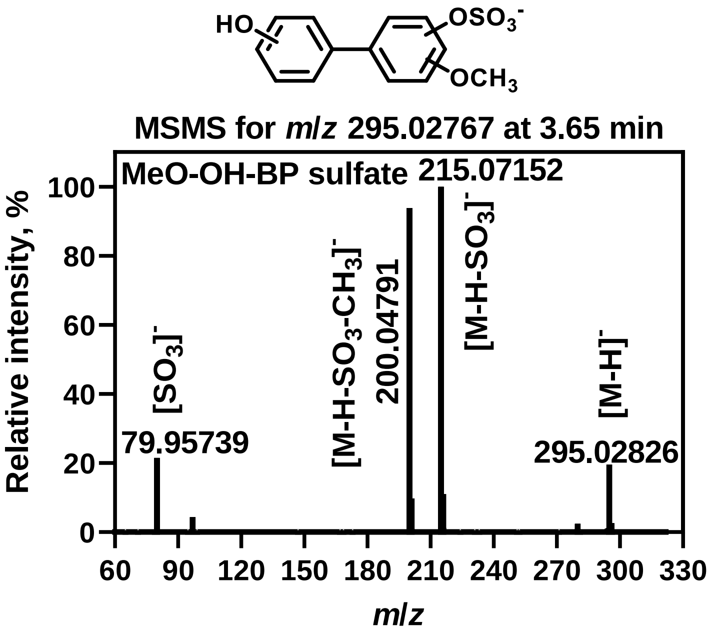

Fig. S12. One MeO-OH-BP sulfate metabolite (eluting at 3.65 min) was present in the cell culture medium from HepG2 cells exposed to 10 µM PCB 2. The identification of this metabolite was supported by the MS/MS spectrum showing fragment ions consistent with MeO-OH-BP sulfate. MS/MS analyses were performed on an LC-Orbitrap MS with an Acquity UPLC BEH C18 column. For the extracted ion chromatogram for MeO-OH-BP sulfate at *m/z* 295.02767, see Fig. 1. For information about the formation of MeO-OH-BP sulfate in the cell culture medium from HepG2 cells exposed to 3.6 nM PCB 2, see Table 1.

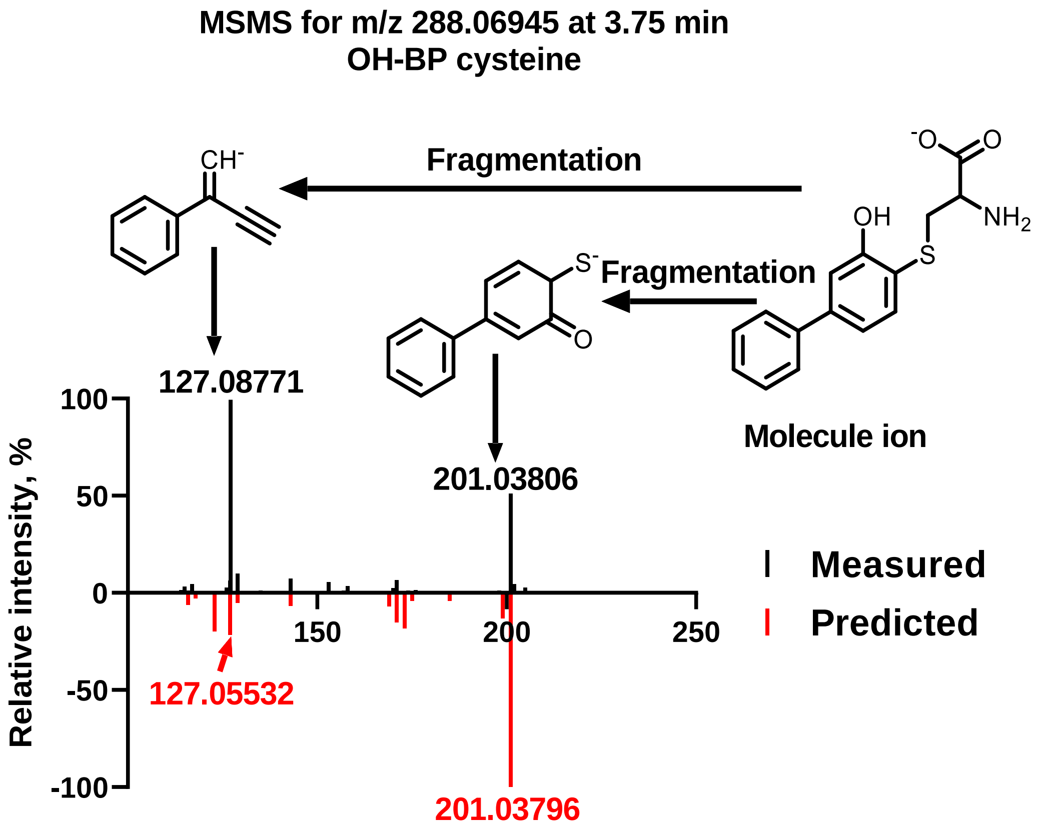

Fig. S13. The agreement of the measured and predicted MS/MS spectra of OH-BP cysteine supports its detection in the cell culture medium from HepG2 cells exposed to 10 µM PCB 2. The MS/MS spectrum was predicted with CFM-ID at 40 eV (collision energy), as described.[27] The MS/MS analysis was performed on an LC-Orbitrap MS with an Acquity UPLC BEH C18 column with a collision energy of 30 eV. For the extracted ion chromatogram of OH-BP cysteine at *m/z* 288.06945, see Fig. 1. For information about the formation of OH-BP cysteine in the cell culture medium from HepG2 cells exposed to 3.6 nM PCB 2, see Table 1.

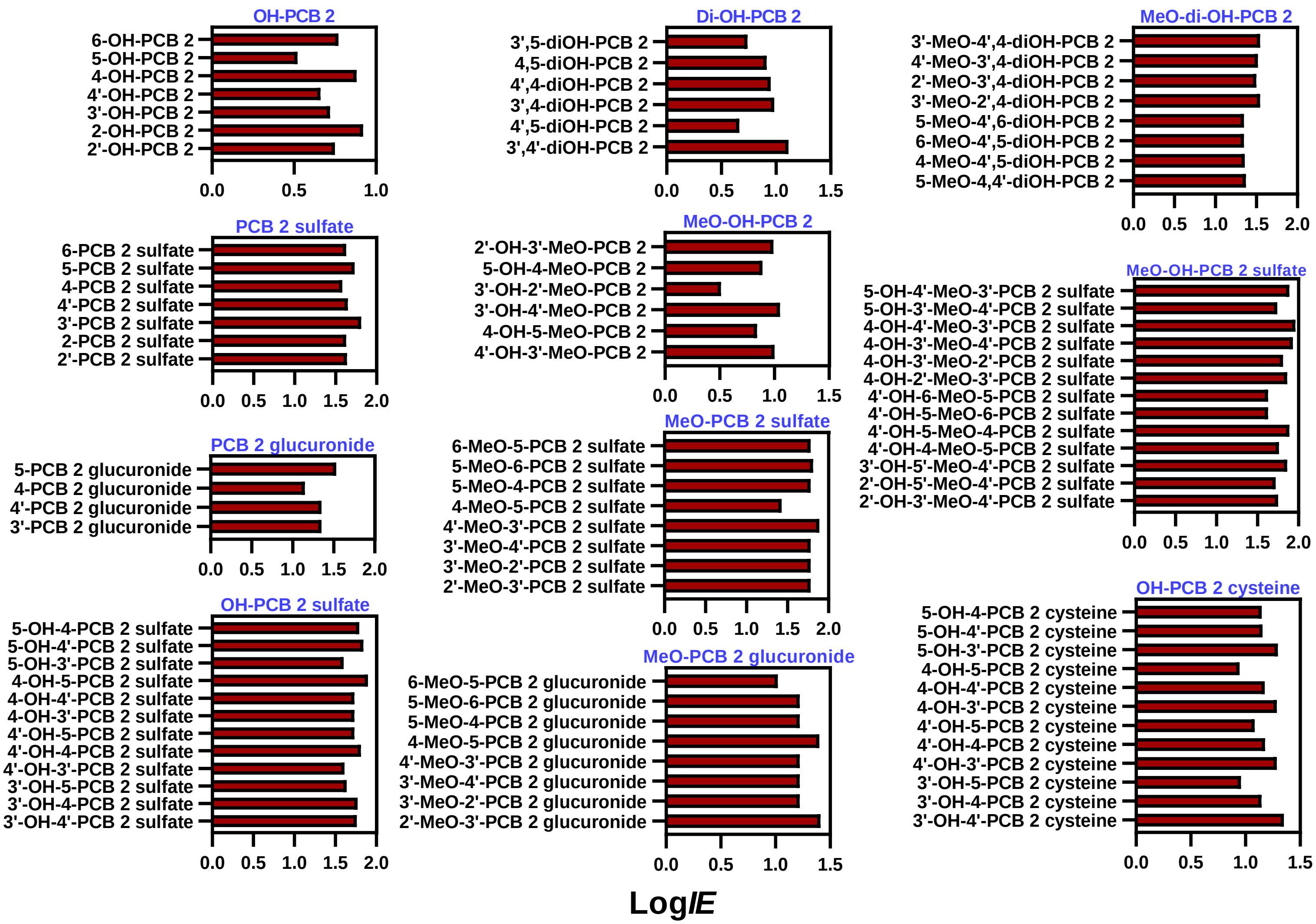

Fig. S14. The logarithmic ionization efficiency values (log*IE*) of eleven classes of PCB 2 metabolites listed in Table 1 and Table S4 were predicted with random forest regression from PaDEL descriptors, as described.[28] In each metabolite class, selected metabolite isomers, mainly substituted at *meta*- or *para* positions, were included in the log*IE* prediction. The log*IE* values were averaged and used for correcting the molecular response in PCB 2 metabolite profiles (Fig. 2).

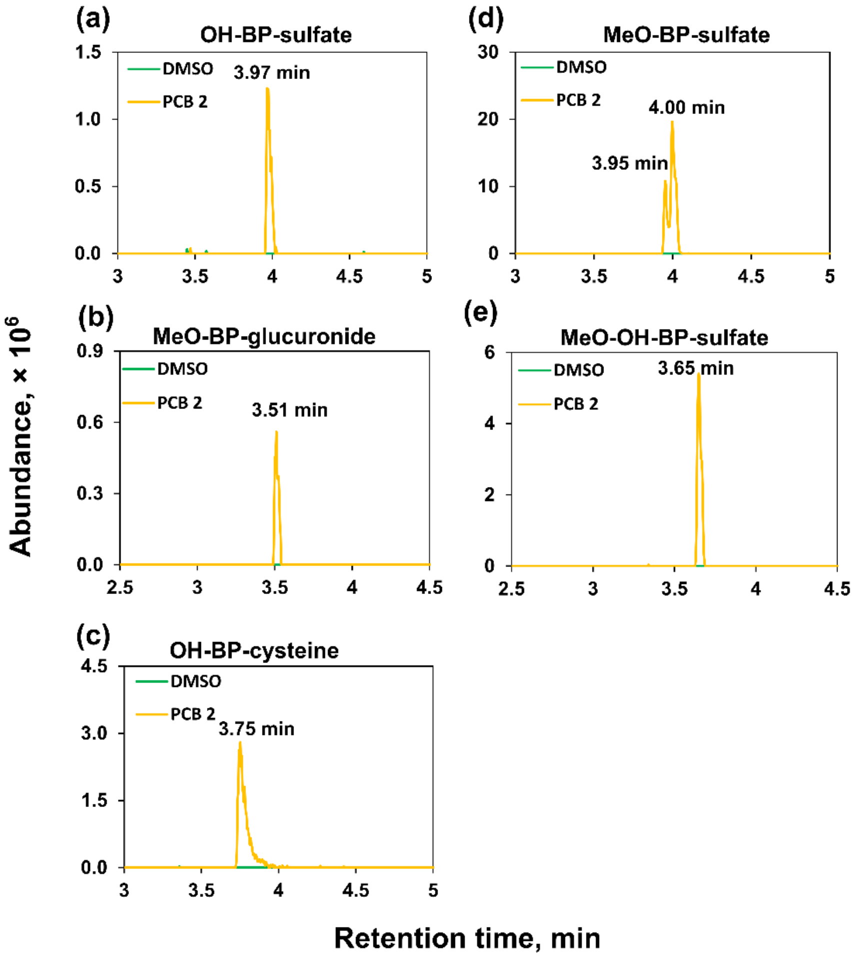

Fig. S15. The formation of BP metabolites from PCB 2 was supported by the clean background levels in the DMSO controls. Chromatograms were extracted at (a) *m/z* 265.01710 for OH-BP sulfate, (b) *m/z* 279.03275 for MeO-BP sulfate, (c) *m/z* 375.10804 for MeO-BP glucuronide, (d) *m/z* 295.02767 for MeO-OH-BP sulfate, and (e) *m/z* 288.06945 for OH-BP cysteine, with a mass window of 10 ppm. Analyses were performed on an LC-Orbitrap MS with an Acquity UPLC BEH C18 column.

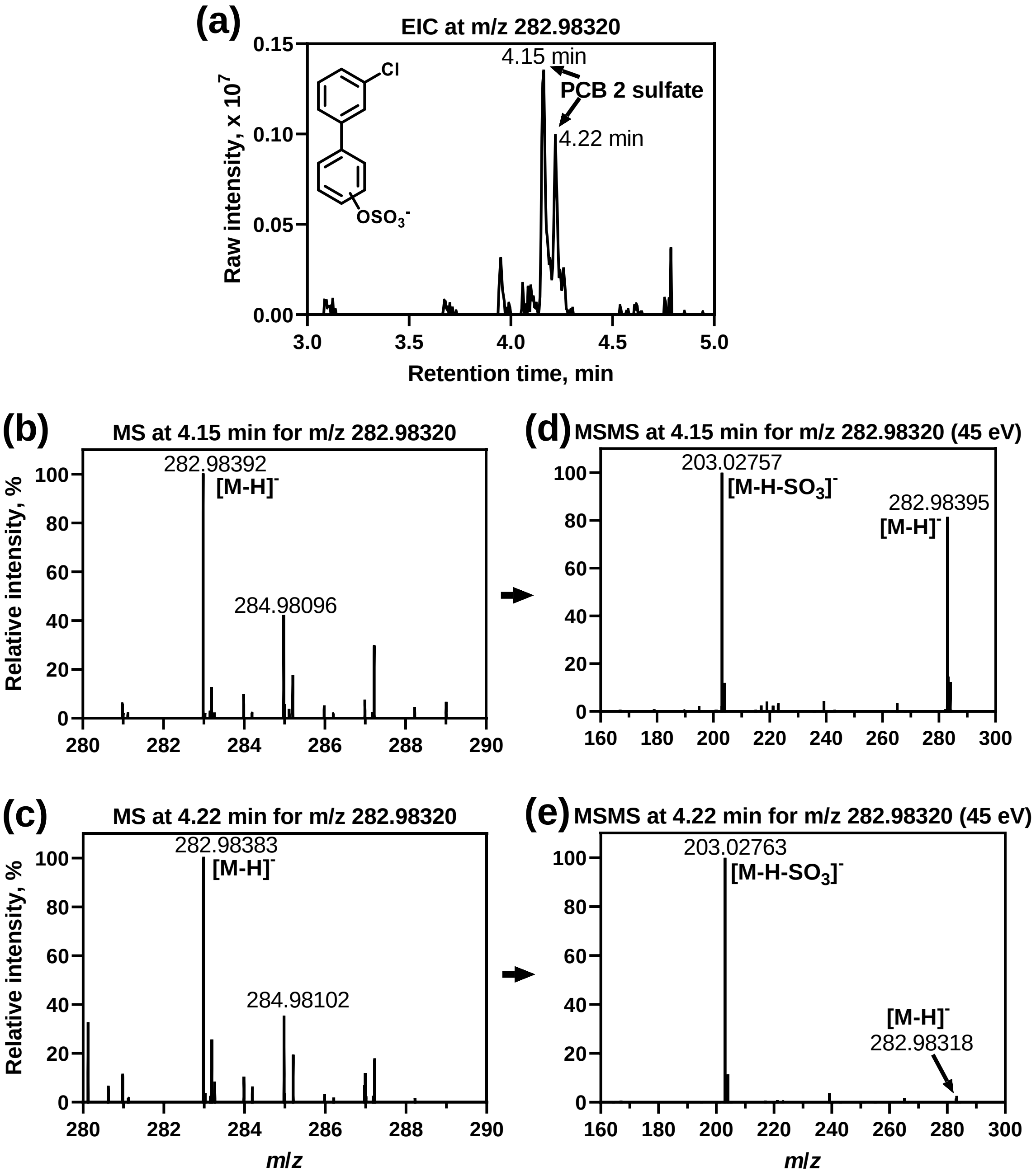

Fig. S16. Two likely PCB 2 sulfate isomers were present in the cell culture medium from HepG2 cells exposed to 10 µM PCB 11. (a) The extracted ion chromatogram (EIC) at *m/z* 282.98320 with a mass window of 10 ppm revealed the presence of two monochlorinated PCB sulfate isomers eluting at 4.15 and 4.22 min. The formation of monochlorinated PCB sulfate metabolites was further confirmed by the accurate mass of the molecular ion and its isotope pattern [panels (b) and (c)]. Furthermore, the MS/MS spectra of both monochlorinated PCB metabolites showed fragment ions consistent with monochlorinated PCB sulfates [panels (d) and (e)]. LC-MS and MS/MS analysis were performed on an LC-Orbitrap MS with an Acquity UPLC BEH C18 column. A detailed description of the metabolism studies with PCB 11 and the identification of the PCB 11 metabolites has been reported previously.[2]

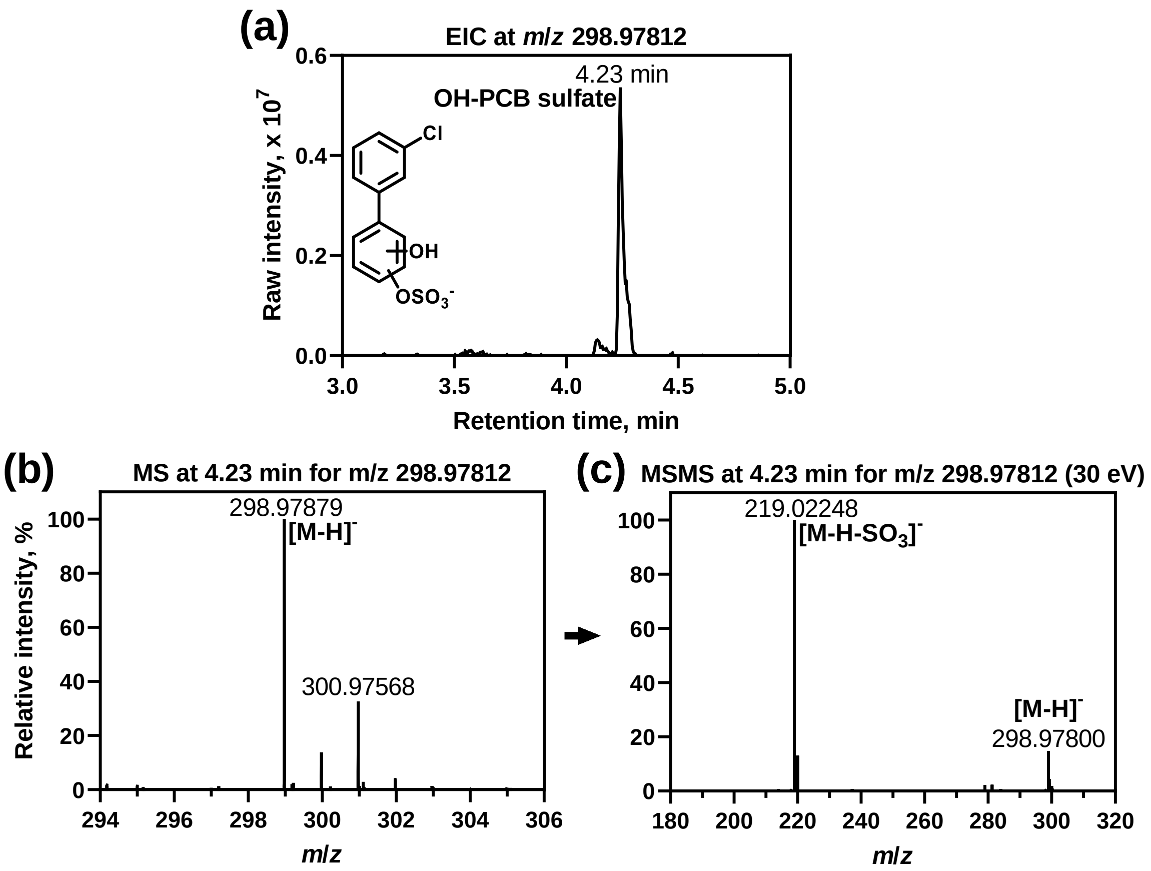

Fig. S17. A putative OH-PCB 2 sulfate was present in the cell culture medium from HepG2 cells exposed to 10 µM PCB 11. (a) The extracted ion chromatogram (EIC) at *m/z* 298.97812 with a mass window of 10 ppm revealed the presence of a monochlorinated OH-PCB sulfate eluting at 4.23 min. The formation of a monochlorinated OH-PCB sulfate metabolite was further confirmed by the accurate mass of the molecular ion and its isotope pattern [panels (b)]. Furthermore, the MS/MS spectra of this metabolite showed fragment ions consistent with a monochlorinated OH-PCB sulfate [panels (c)]. LC-MS and MS/MS analysis were performed on an LC-Orbitrap MS with an Acquity UPLC BEH C18 column. A detailed description of the metabolism studies with PCB 11 and the identification of the PCB 11 metabolites has been reported previously.[2]

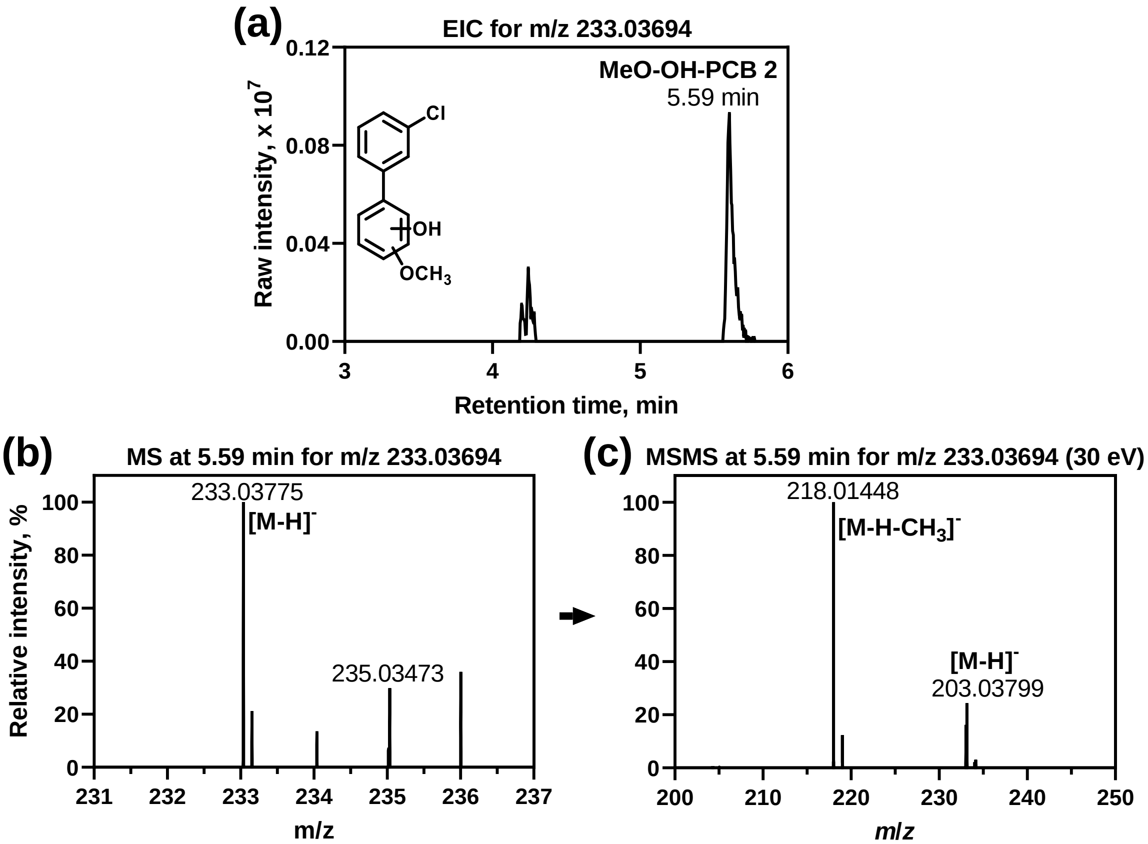

Fig. S18. A putative MeO-OH-PCB 2 metabolite was present in the cell culture medium from HepG2 cells exposed to 10 µM PCB 11. (a) The extracted ion chromatogram (EIC) at *m/z* 233.03694 with a mass window of 10 ppm revealed the presence of a monochlorinated MeO-OH-PCB metabolite eluting at 5.59 min. The formation of a monochlorinated MeO-OH-PCB metabolite was further confirmed by the accurate mass of the molecular ion and its isotope pattern [panels (b)]. Furthermore, the MS/MS spectra of this metabolite showed fragment ions consistent with a monochlorinated MeO-OH-PCB metabolite [panels (c)]. LC-MS and MS/MS analysis were performed on an LC-Orbitrap MS with an Acquity UPLC BEH C18 column. A detailed description of the metabolism studies with PCB 11 and the identification of the PCB 11 metabolites has been reported previously.[2]

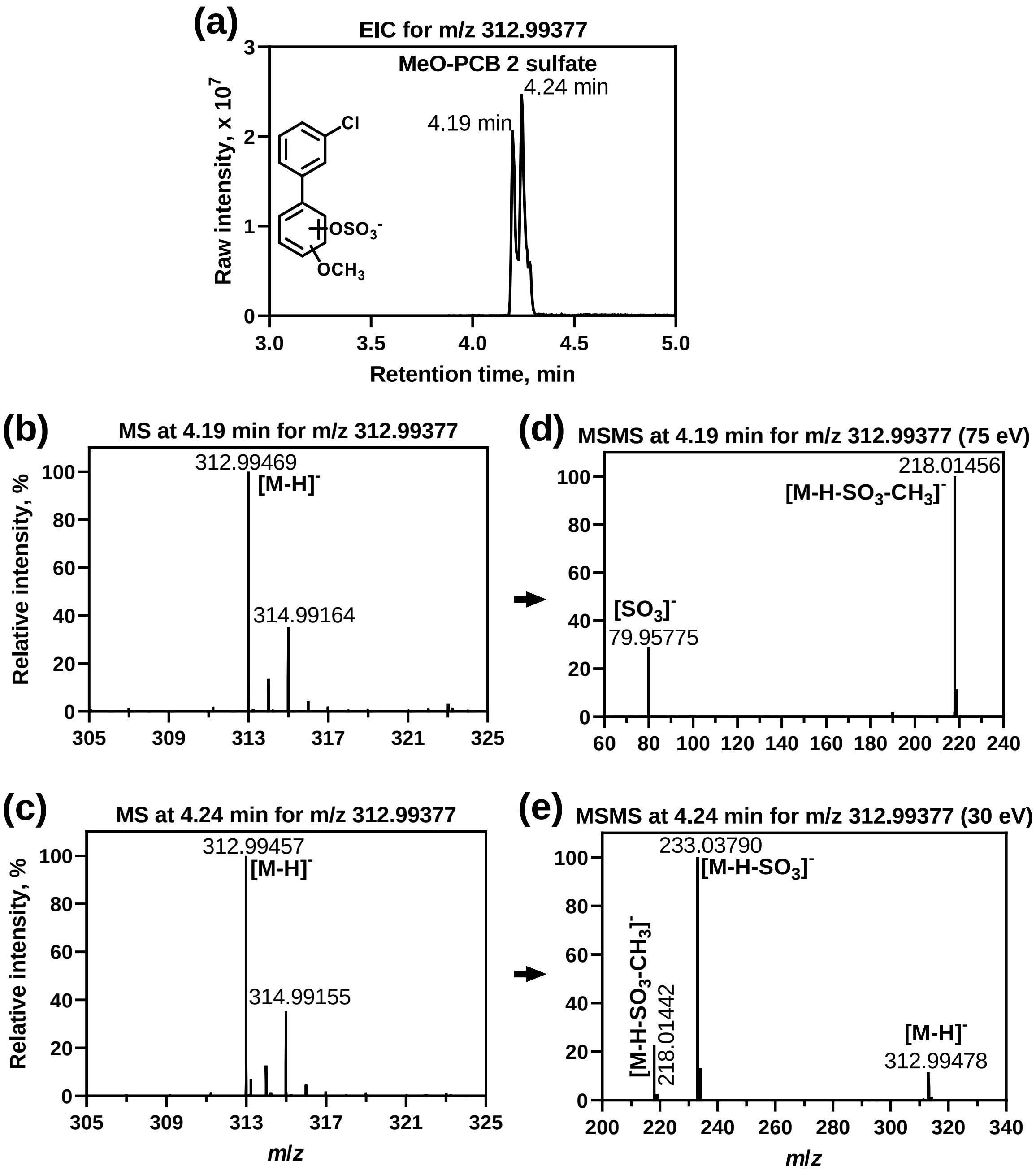

Fig. S19. Two putative MeO-PCB 2 sulfate isomers were present in the cell culture medium from HepG2 cells exposed to 10 µM PCB 11. (a) The extracted ion chromatogram (EIC) at *m/z* 312.99377 with a mass window of 10 ppm revealed the presence of two monochlorinated MeO-PCB sulfate isomers eluting at 4.19 and 4.24 min. The formation of monochlorinated MeO-PCB sulfate metabolites was further confirmed by the accurate mass of the molecular ion and its isotope pattern [panels (b) and (c)]. Furthermore, the MS/MS spectra of both metabolites showed fragment ions consistent with monochlorinated MeO-PCB sulfates [panels (d) and (e)]. LC-MS and MS/MS analysis were performed on an LC-Orbitrap MS with an Acquity UPLC BEH C18 column. A detailed description of the metabolism studies with PCB 11 and the identification of the PCB 11 metabolites has been reported previously.[2]

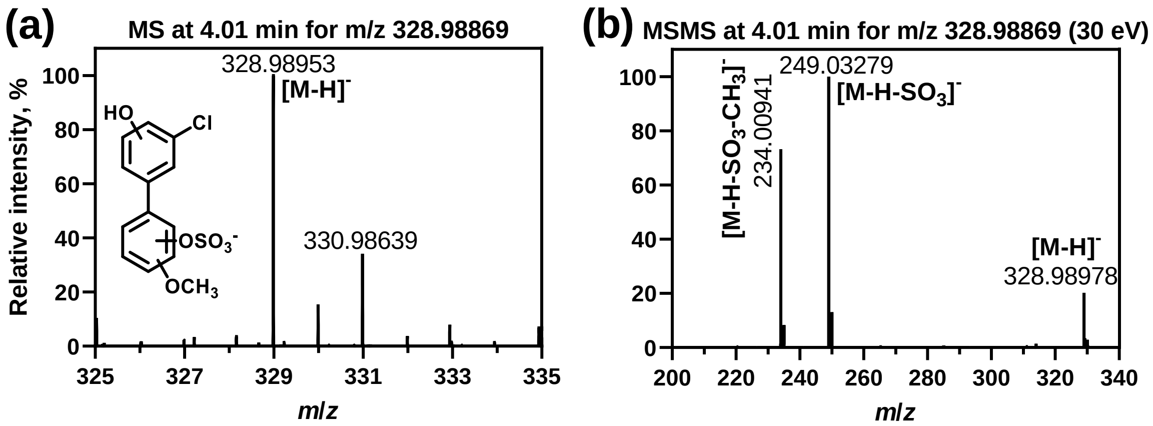

Fig. S20. A putative MeO-OH-PCB 2 sulfate metabolite (eluting at 4.01 min) was present in the cell culture medium from HepG2 cells exposed to 10 µM PCB 11. The identification of this metabolite was supported by (a) the MS spectrum showing the accurate mass of the molecular ion and its isotope pattern and (b) the MS/MS spectra showing fragment ions consistent with a monochlorinated MeO-OH-PCB sulfate. LC-MS and MS/MS analysis were performed on an LC-Orbitrap MS with an Acquity UPLC BEH C18 column. For the extracted ion chromatogram and the MS/MS spectrum of a second putative MeO-OH-PCB 2 sulfate at *m/z* 328.98869 (eluting 3.97 min), see Fig. 4. A detailed description of the metabolism studies with PCB 11 and the identification of the PCB 11 metabolites has been reported previously.[2]

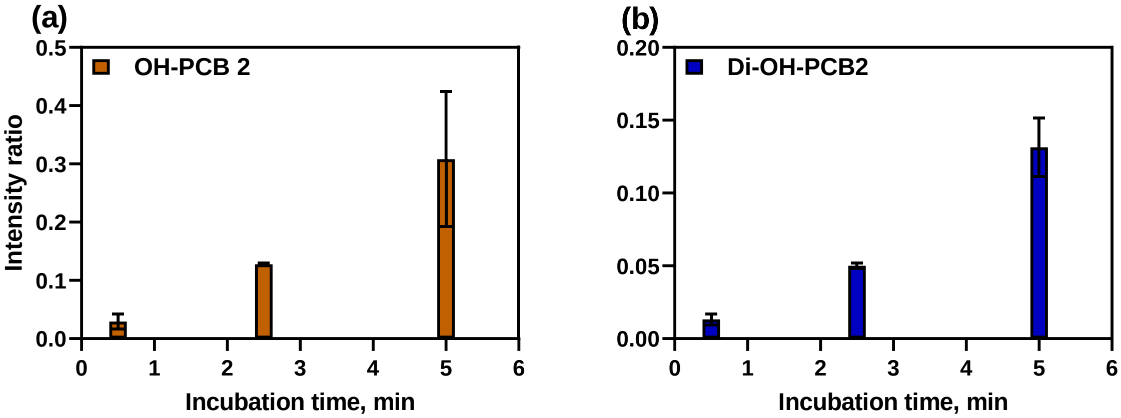

Fig. S21. The time course of the formation of (a) OH-PCB 2 and (b) di-OH-BP in HLM incubations with 10 μM PCB 2. The intensity ratios were calculated as the intensity at each timepoint/the intensity at the last time point (i.e., 15 min). Error bars were calculated from two independent experiments. The raw intensities were collected from extracted ion chromatograms at the theoretical accurate mass with a time window of 10 ppm. For the chromatograms showing the formation of OH-PCB 2 and Di-OH-PCB 2 in the HLM incubations, see Fig. 3.

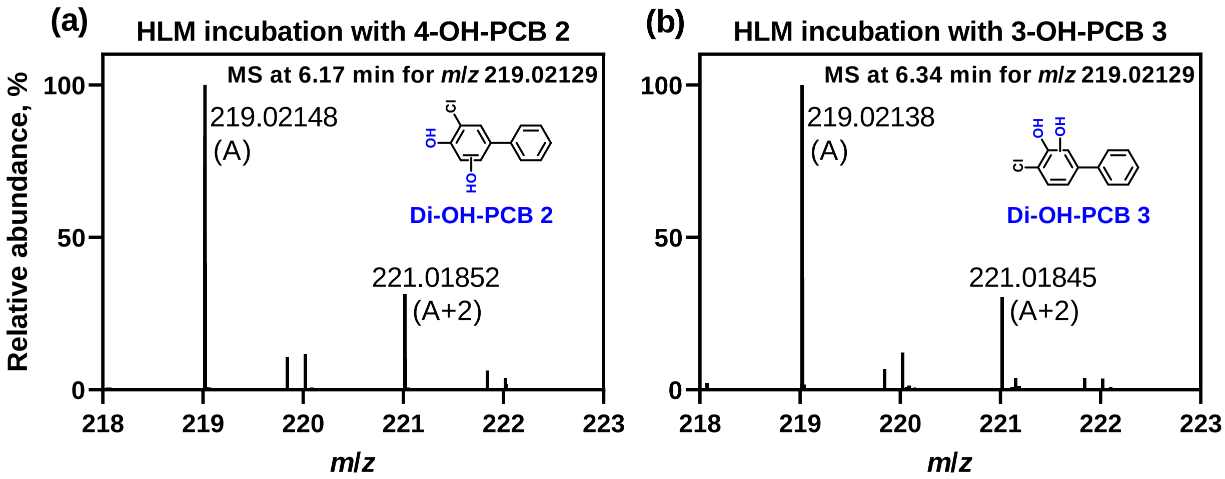

Fig. S22. The identification of di-OH-PCB metabolites was supported by MS spectra showing the accurate mass of the molecular ion and their isotope patterns for metabolites eluting at (a) 6.17 min and (b) 6.34 min in the HLM incubations with 10 μM 4-OH-PCB 2 and 3-OH-PCB 3, respectively. LC-MS analyses were performed on an LC-Orbitrap MS with an Acquity UPLC BEH C18 column. For the extracted ion chromatogram for di-OH-PCB 2 and di-OH-PCB3 metabolites at m/z 312.99377, see Fig. 3.

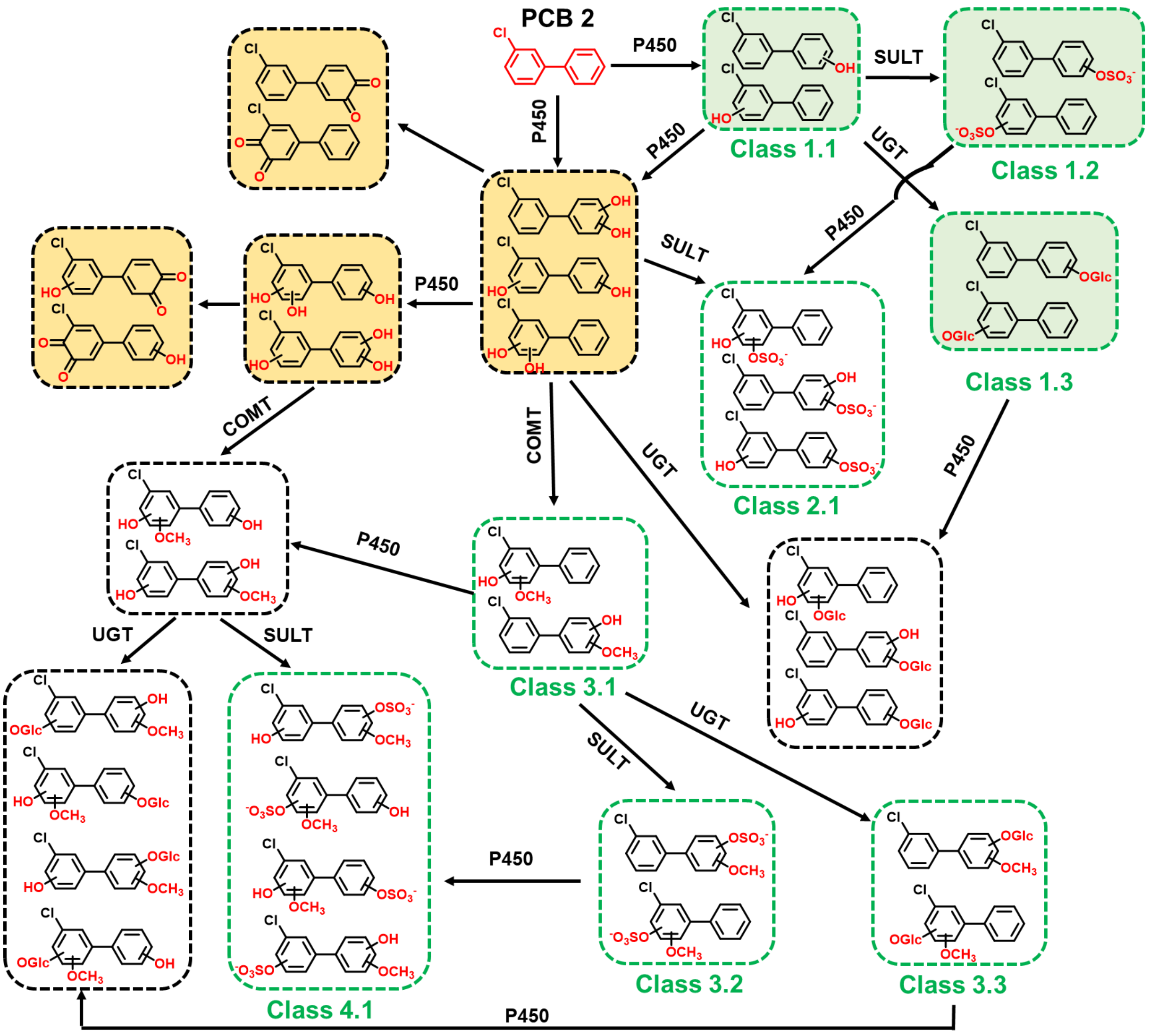

Fig. S23. Metabolism scheme showing the complex metabolism of PCB 2 in HepG2 cells. Structures shown in black boxes were not detected in this study, but their formation is expected based on this and other studies. Catechol metabolites and their quinone derivatives are shown on an orange background. These metabolites are likely reactive and highly toxic. MetaDrug predicted structures on a light green background. The placement of the functional groups is for illustration purposes only and does not indicate their actual position. P450: Cytochrome P450 enzyme; Glc: Glucuronide; SULT: Sulfotransferase; UGT: Uridine 5'-diphospho-glucuronosyltransferase; COMT: Catechol-O-methyltransferase

References

1. Zhang, C.-Y.; Flor, S.; Ruiz, P.; Ludewig, G.; Lehmler, H.-J. Characterization of the metabolic pathways of 4-chlorobiphenyl (PCB3) in HepG2 cells using the metabolite profiles of its hydroxylated metabolites. *Environ. Sci. Technol.* **2021,** *55*, 9052-9062.
2. Zhang, C.-Y.; Flor, S.; Ruiz, P.; Dhakal, R.; Hu, X.; Teesch, L. M.; Ludewig, G.; Lehmler, H.-J. 3,3'-Dichlorobiphenyl is metabolized to a complex mixture of oxidative metabolites, including novel methoxylated metabolites, by HepG2 cells. *Environ. Sci. Technol.* **2020,** *54*, 12345-12357.
3. Kania-Korwel, I.; Lehmler, H. J. Chiral polychlorinated biphenyls: absorption, metabolism and excretion-a review. *Environ. Sci. Pollut. Res.* **2016,** *23*, 2042-2057.
4. Grimm, F. A.; Hu, D. F.; Kania-Korwel, I.; Lehmler, H. J.; Ludewig, G.; Hornbuckle, K. C.; Duffel, M. W.; Bergman, A.; Robertson, L. W. Metabolism and metabolites of polychlorinated biphenyls. *Crit. Rev. Toxicol.* **2015,** *45*, 245-272.
5. Uwimana, E.; Ruiz, P.; Li, X. S.; Lehmler, H. J. Human CYP2A6, CYP2B6, AND CYP2E1 atropselectively metabolize polychlorinated biphenyls to hydroxylated metabolites. *Environ. Sci. Technol.* **2019,** *53*, 2114-2123.
6. Kaminsky, L. S.; Kennedy, M. W.; Adams, S. M.; Guengerich, F. P. Metabolism of dichlorobiphenyls by highly purified isozymes of rat-liver cytochrome-P-450. *Biochemistry* **1981,** *30*, 577-588.
7. Warner, N. A.; Martin, J. W.; Wong, C. S. Chiral polychlorinated biphenyls are biotransformed enantioselectively by mammalian cytochrome P-450 isozymes to form hydroxylated metabolites. *Environ. Sci. Technol.* **2009,** *43*, 114-121.

8. Lu, Z.; Kania-Korwel, I.; Lehmler, H. J.; Wong, C. S. Stereoselective formation of mono- and dihydroxylated polychlorinated biphenyls by rat cytochrome P450 2B1. *Environ. Sci. Technol.* **2013,** *47*, 12184-12192.

9. McGraw, J. E.; Waller, D. P. Specific human CYP 450 isoform metabolism of a pentachlorobiphenyl (PCB-IUPAC# 101). *Biochem. Biophs. Res. Commun.* **2006,** *344*, 129-133.

10. Shimada, T.; Kakimoto, K.; Takenaka, S.; Koga, N.; Uehara, S.; Murayama, N.; Yamazaki, H.; Kim, D.; Guengerich, F. P.; Komori, M. Roles of human CYP2A6 and monkey CYP2A24 and 2A26 cytochrome P450 enzymes in the oxidation of 2,5,2',5'-tetrachlorobiphenyl. *Drug Metab. Dispos.* **2016,** *44*, 1899-1909.

11. Kennedy, M. W.; Carpentier, N. K.; Dymerski, P. P.; Kaminsky, L. S. Metabolism of dichlorobiphenyls by hepatic-microsomal cytochrome-P-450. *Biochem. Pharmacol.* **1981,** *30*, 577-588.

12. Uwimana, E.; Li, X. S.; Lehmler, H. J. Human liver microsomes atropselectively metabolize 2,2 ',3,4 ',6-pentachlorobiphenyl (PCB 91) to a 1,2-shift product as the major metabolite. *Environ. Sci. Technol.* **2018,** *52*, 6000-6008.

13. Daidoji, T.; Gozu, K.; Iwano, H.; Inoue, H.; Yokota, H. UDP-glucuronosyltransferase isoforms catalyzing glucuronidation of hydroxy-polychlorinated biphenyls in rat. *Drug Metab. Dispos.* **2005,** *33*, 1466-1476.

14. Dhakal, K.; He, X. R.; Lehmler, H. J.; Teesch, L. M.; Duffel, M. W.; Robertson, L. W. Identification of sulfated metabolites of 4-chlorobiphenyl (PCB3) in the serum and urine of male rats. *Chem. Res. Toxicol.* **2012,** *25*, 2796-2804.

15. Hu, X.; Lehmer, H. J.; Adamcakova-Dodd, A.; Thorne, P. S. Elimination of inhaled 3,3 '-dichlorobiphenyl and the formation of the 4-hydroxylated metabolite. *Environ. Sci. Technol.* **2013,** *47*, 4743-4751.

16. Tulp, M. T. M.; Bruggeman, W. A.; Hutzinger, O. Reductive dechlorination of chlorobiphenylols by rats. *Experientia* **1977,** *33*, 1134-1136.

17. Safe, S.; Jones, D.; Hutzinger, O. Metabolism of 4,4'-dihalogenobiphenyls. *J. Chem. Soc. Perkin Trans. 1* **1976**, 357-359.

18. Tulp, M. T. M.; Sundström, G.; Hutzinger, O. The metabolism of 4,4'-dichlorobiphenyl in rats and frogs. *Chemosphere* **1976,** *5*, 425-432.

19. Hutzinger, O.; Jamieson, W. D.; Safe, S.; Paulmann, L.; Ammon, R. Identification of metabolic dechlorination of highly chlorinated biphenyl in rabbit. *Nature* **1974,** *252*, 698-699.

20. Schymanski, E. L.; Jeon, J.; Gulde, R.; Fenner, K.; Ruff, M.; Singer, H. P.; Hollender, J. Identifying small molecules via high resolution mass spectrometry: communicating confidence. *Environ. Sci. Technol.* **2014,** *48*, 2097-2098.

21. Harris, D. C., *Quantitative Chemical Analysis*. W.H. Freeman and Company: New York, 2010.

22. Li, X.; Holland, E. B.; Feng, W.; Zheng, J.; Dong, Y.; Pessah, I. N.; Duffel, M. W.; Robertson, L. W.; Lehmler, H. J. Authentication of synthetic environmental contaminants and their (bio)transformation products in toxicology: polychlorinated biphenyls as an example. *Environ. Sci. Pollut. Res.* **2018,** *25*, 16508-16521.

23. Holland, E. B.; Feng, W.; Zheng, J.; Dong, Y.; Li, X. S.; Lehmler, H. J.; Pessah, I. N. An extended structure-activity relationship of nondioxin-like PCBs evaluates and supports

modeling predictions and identifies picomolar potency of PCB 202 towards ryanodine receptors. *Toxicol. Sci.* **2017,** *155*, 170-181.

24. Wishart, D. S.; Jewison, T.; Guo, A. C.; Wilson, M.; Knox, C.; Liu, Y. F.; Djoumbou, Y.; Mandal, R.; Aziat, F.; Dong, E.; Bouatra, S.; Sinelnikov, I.; Arndt, D.; Xia, J. G.; Liu, P.; Yallou, F.; Bjorndahl, T.; Perez-Pineiro, R.; Eisner, R.; Allen, F.; Neveu, V.; Greiner, R.; Scalbert, A. HMDB 3.0-The human metabolome database in 2013. *Nucleic Acids Res.* **2013,** *41*, D801-D807.
25. Kanehisa, M.; Furumichi, M.; Tanabe, M.; Sato, Y.; Morishima, K. KEGG: new perspectives on genomes, pathways, diseases and drugs. *Nucleic Acids Res.* **2017,** *45*, D353-D361.
26. Uppal, K.; Walker, D. I.; Jones, D. P. xMSannotator: An R package for network-based annotation of high-resolution metabolomics data. *Anal. Chem.* **2017,** *89*, 1063-1067.
27. Allen, F.; Pon, A.; Wilson, M.; Greiner, R.; Wishart, D. CFM-ID: a web server for annotation, spectrum prediction and metabolite identification from tandem mass spectra. *Nucleic Acids Res.* **2014,** *42*, W94-W99.
28. Liigand, J.; Wang, T. T.; Kellogg, J.; Smedsgaard, J.; Cech, N.; Kruve, A. Quantification for non-targeted LC/MS screening without standard substances. *Sci. Rep.* **2020,** *10*, 5808.
